# HAttFFNN: Hybridized attention mechanism-based feedforward neural network deep learning model for the plastic material classification of three stage materials on spectroscopic data

**DOI:** 10.1371/journal.pone.0336927

**Published:** 2025-12-02

**Authors:** Nazym Alimbekova, Hari Mohan Rai, Tursinbay Turymbetov, Ainur Zhumadillayeva

**Affiliations:** 1 Department of Computer and Software Engineering, L.N. Gumilyov Eurasian National University, Astana, Kazakhstan; 2 High School of Information Technology and Engineering, Astana, Kazakhstan; 3 Department of Computer Science, School of Engineering and Digital Sciences, Nazarbayev University, Astana, Kazakhstan; 4 Humanities school, International University of Tourism and Hospitality, Turkistan, Kazakhstan; Al-Nahrain University, IRAQ

## Abstract

Classification of plastic materials based on spectroscopic data is a very crucial task in a variety of applications, including automated recycling, environmental monitoring, quality control in manufacturing, quality control of products, and analysis of complex material properties. These applications demand high precision in identifying and separating plastic types to enhance sustainability and ensure regulatory compliance. In this work, we presented a novel technique Hybridized Attention mechanism-based Feedforward Neural Network (HAttFFNN) to detect three stage Polyethylene Terephthalate (PET) materials. Dataset used in this methodology is basically comprised of 295,327 samples, and contains the parameters like absorbance, wavelengths, references, samples. We collected the spectral data (900–1700 nm) using the Digital Light Processing (DLP) Near-Infrared (NIR) scan Nano Evaluation Module (EVM). We utilized various preprocessing techniques for better and improved detection result, such as Savitzky-Golay filter, interference, Standard Normal Variate (SNV) and Multiplicative Scatter Correction (MSC). The preprocessed and organized spectral data is provided to the proposed HAttFFNN model for the detection of three stage PET material. To validate the performance of the proposed model, we experimented various State-Of-The-Art (SOTA) models, Multi-Head Neural Network (MHNN), Virtual Geometry Group (VGG16), One-Dimensional Convolutional Neural Network (1D-CNN), Residual Network (ResNet), Long Short-Term Memory (LSTM) and Gated Recurrent Unit (GRU). The proposed model outperforms state of the art techniques across all metrics including accuracy, precision, recall, F1 score, and specificity with Stage 1 (PET Clear vs PET Hazard) achieving 99.33% accuracy, Stage 2 (PET vs Others) 99.32%, and Stage 3 (PET Coloured vs PET Transparent) 99.28%, along with consistently high precision, recall, and specificity values for each class. These results confirm that our proposed model, HAttFFNN, is able to achieve higher accuracy in spectroscopic classification domain, especially in complex cases such as differentiating between visually and spectrally similar materials (PET Clear vs PET Hazard, PET vs Others and PET Colored vs PET Transparent) where traditional models often fail. Furthermore, the Root Mean Square Error (RMSE) values 0.1408 for Stage 1, 0.1249 for Stage 2, and 0.1403 for Stage 3, further validate the model’s low-error performance, reinforcing its effectiveness as a less error-prone approach for spectrometry-based plastic material classification.

## 1. Introduction

The huge amount of waste materials is generated every day, especially plastic based waste materials which are hazardous to the environment [1]. These hazardous waste materials create major issues in the numerous cities and their living humans and pet animals [2]. The toxic hazards present in the waste materials create variety of issues such as health issues and environmental issues [3]. The plastic bottles are the most hazardous material present in these wastes [4]. The plastic bottles are non-biodegradable and they cannot be destroyed by any means, hence they create huge impact on the environment [5]. The best way to overcome these issues to recycle the waste materials without damaging the environment. Hence, the types of materials need to be identified before utilizing it for the recycling process [6,7]. Sorting of the plastic materials are the critical task as they are found to be in various categories, sizes, colors, and shapes [8]. Polyethylene Terephthalate (PET) is one of the most commonly available material found in the plastic bottles [9,10]. Spectroscopic-based material identification is considered to be one of the most important tasks in different fields such as recycling, quality control in industries, and evaluation of the properties of multi-component materials [11]. The need for fast and highly accurate material classification systems has risen due to enhancements in materials environmental classification, industrial automation, and intelligent manufacturing systems [12]. In particular, PET is used in packaging and consumer products industries and thus the effective sortation of PET is important for recycling and reusing purposes [13]. Most methodologies of classification are normally based on a manual estimation of spectroscopic information, which is very laborious and inaccurate [14]. Deep learning techniques when combined with spectroscopic data analysis provides a very effective and sustainable solution to all these issues with effective and almost hundred percent classification [15]. The dataset applied in this study contains 295,327 samples and contains features such as wavelength, absorbance, reference signals, sample signals, and category-specific characteristics [16,17]. These features can be used as a basis for differentiating between PET clear, PET other and transparent materials [18]. The major problem still arises from obtaining high accuracy while maintaining high levels of robustness to spectroscopic data noise and small measurement fluctuations that are still present in today’s machine learning algorithms, even though there are more complex and sophisticated models readily available [19].

Although many traditional ML and DL models have been applied for the plastic material classification tasks, but they generally suffer from the notable drawbacks such as sensitivity to spectral noise, poor generalization across heterogeneous plastic categories, and also face difficulty in handling the overlapping spectral features. These challenges limit their effectiveness in the real-world recycling applications, where data variability is high and class boundaries are not always distinct. Therefore, the proposed method aims to overcome these limitations by introducing a robust, attention-enhanced architecture which is tailored to improve the classification accuracy and resilience across diverse spectroscopic conditions.

The main contributions in the paper are highlighted as follows:

- Proposed Hybridized Attention mechanism-based Feedforward Neural Network (HAttFFNN) for the classification of three stage PET materials.
- Utilized big dataset containing 295,327 data samples, containing various critical parameters such as, absorbance, wavelengths, references, samples, and category properties.
- Collected the spectral data in the 900–1700 nm range using DLP-NIR scan Nano Evaluation Module (EVM) ensuring high quality spectroscopic information.
- Utilized advanced preprocessing techniques including Savitzky-Golay filtering, interference correction, Standard Normal Variate (SNV), and Multiplicative Scatter Correction (MSC).
- Utilized three stage classification of the plastic materials, stage 1: PET Clear vs PET Hazard, stage 2: PET vs Others, and Stage 3: PET Colored vs PET Transparent.
- Performance evaluation against several SOTA models, VGG16, Multi-Head Neural Network, ResNet, 1D-CNN, LSTM, and GRU, across multiple performance metrics (accuracy, precision, recall, F1-score, and specificity).

This paper is structured as follows: Section 2 reviews related work in spectroscopic data analysis and deep learning methods. Section 3 details the materials and methods section which also includes proposed multi-head network architecture and data preprocessing techniques. Section 4 presents the experimental setup, results, and performance analysis, followed by a discussion. Finally, Section 5 concludes the paper with potential directions for future research.

### 2. Related works

Table 1 shows a list of research works that aim to identify the material, especially plastics, using spectroscopic information and ML algorithms. The above-mentioned articles also use various ML techniques like CNN for recycling, Random Forest, SVM, LSTM for better material recognition for recycled materials and quality assurance. Among the outcomes, it has been pointed out that current deep learning methods assist material classification tasks, and they outperform previous approaches in terms of accuracy. Some of the limitations highlighted in the studies include use of limited and different models, presence of noisy data and requirement of large datasets so that overfitting is avoided. Some of the methods such as Random Forest and SVM as previously seen may perform well in a particular setting but are not very good at dealing with large data or noisy spectral data. However, more sophisticated models like CNNs and LSTMs which are very efficient have their limitations such as relatively large computational cost, large requirement for training data and also need to undergo thorough preprocessing to make them functional in realistic environments. The existing literature also shows the attempts of enhancing these methods and to consider new opportunities for advancing the techniques of material classification with the focus more on sustainability and recycling.

**Table 1:** Literature review on material classification and spectroscopic data analysis.

Literature	Purpose	Method	Key Findings	Challenges
[20]	Classify materials based on spectroscopic data	CNN	Achieved 95% accuracy in material classification using spectral data	Limited generalizability to other material types
[21]	Improve recycling efficiency using machine learning and spectroscopy	Random Forest, PCA	Successfully identified different polymer types for recycling with high precision	Difficulty in handling high-dimensional data
[22]	Classify plastic materials in waste management	Support Vector Machine (SVM)	SVM outperformed traditional methods for identifying PET and other plastic types	SVM’s performance reduced with noisy data
[23]	Identify materials in industrial processes for quality control	ANN (Artificial Neural Networks)	ANN model showed significant improvement over traditional methods in identifying plastic types	Limited sample diversity and model overfitting
[24]	Investigate spectral data for efficient material categorization in recycling	Convolutional Neural Networks (CNN)	CNN model achieved excellent classification accuracy, making it suitable for material identification	Needs extensive data preprocessing for real-world applications
[25]	Classify polymer materials for sustainable recycling efforts	LSTM (Long Short-Term Memory Networks)	LSTM models provided good performance in identifying different plastics, contributing to better recycling processes	LSTM networks are complex and require large training data
[26]	Develop an automated system for plastic sorting based on spectroscopic signals	Deep Learning (CNN + RNN Hybrid)	A hybrid CNN and RNN approach improved the accuracy of material classification	The system needs optimization for real-time deployment
[27]	Predict material types using Raman spectroscopic data	XGBoost and Decision Trees	XGBoost showed high performance in identifying material categories with minimal preprocessing	Insensitivity to subtle material variations in spectroscopic data
[28]	Improve quality control for plastic sorting in industrial environments	Neural Networks and Feature Selection	Neural networks with feature selection outperformed traditional methods in identifying polymer types	Overfitting due to high dimensionality of input features
[29]	To classify and identify microplastics using Raman spectroscopy and deep learning	Recorded Raman spectra of 8 plastics; applied LDA, Decision Tree, SVM, and 1D-CNN	1D-CNN achieved 97% accuracy; showed Raman + DL is effective for microplastic classification	Requires optimal acquisition parameters and effective noise reduction
[30]	To identify types of plastic ingested by seabirds	Raman and infrared spectroscopy on 246 ingested items; machine learning for classification	92% confirmation of plastic items; 98% were LD polymers; ML achieved 93% accuracy	Biological contamination hinders conventional library-based identification
[31]	To recognize household plastic waste using low-cost spectroscopy sensor and ML	Multi-spectral sensor capturing 18 wavelengths; applied 10 ML algorithms	CNN and MLP showed best accuracy (83.5% max for PS); sensor is affordable and portable	Lower accuracy for some plastics (e.g., 66% for PET); limited spectral resolution
[32]	To detect microplastics (MPs) in soil efficiently	Used VNIR, InGaAs, and MCT hyperspectral systems + ML	MCT and InGaAs systems achieved up to 100% accuracy in detecting MPs in soil	VNIR had low accuracy (44–87%); small MP concentrations are hard to distinguish
[33]	To evaluate handheld devices for plastic identification	Compared Plastic Scanner, SpectraPod, and high-res system; applied SVM, RF, XGBoost, GNB	High-res: 97% accuracy; SpectraPod: 93%; Scanner: 70%	Handheld devices limited by spectral range and LED sensitivity
[34]	To apply HSI and ML in realistic plastic litter detection	HSI (900–1700 nm) with mRMR, PCA, LDA, KNN in lab and outdoor settings	MCC > 0.94 indoors; > 0.90 outdoors; good transferability of indoor-trained models	Shape of samples affects MCC (range 0.48–0.96); outdoor lighting may affect results
[35]	To enhance identification of food-packaging plastics using NIR and THz spectroscopy	Combined NIR, THz spectroscopy, XGBoost, Bayesian optimization, XAI	>90% precision; different THz frequencies key to differentiating PET and PS	Spectral variability due to additives and shapes; complex interpretation of THz
[36]	To classify and sort flexible plastic packaging waste	HSI (SWIR: 1000–2500 nm) + Hi-PLS-DA model with PCA preprocessing	87.5% accuracy; 98.2% recovery rate; 94.4% purity	Misclassification in filaments and multilayer films; need FTIR validation

Despite notable progress in spectroscopic-based plastic classification, existing methodologies still face several key limitations. One major issue is their limited generalization capability, especially when dealing with contaminated, low-signal, or compositionally similar materials such as clear versus semi-transparent PET. Additionally, many models demonstrate high sensitivity to spectral noise and tend to perform poorly with colored or black plastics. Most current approaches rely on narrow preprocessing pipelines and utilize shallow architectures that lack comprehensive attention-based mechanisms to optimize learning across complex spectral features and temporal sequences. Recognizing this gap, we propose Hybridized Attention mechanism-based Feedforward Neural Network (HAttFFNN), a deep learning model tailored for plastic material classification across three critical stages using spectroscopic data. This holistic framework is specifically designed to handle noisy and heterogeneous near-infrared (NIR) data, effectively compensating for low spectral contrast and ensuring robust performance across diverse material variations, thereby advancing the current state of spectroscopic plastic classification.

## 3. Materials and methods

The dataset used in this study consists of 295,327 samples of spectroscopic data, each with detailed measurements such as absorbance, wavelength, reference signals, sample signals, and category labels, which correspond to various types of plastics and their specific attributes. To ensure robust classification performance, the data were duplicated to create three distinct binary classification tasks: PET clear, PET other and transparent colored..

**Fig 1:**
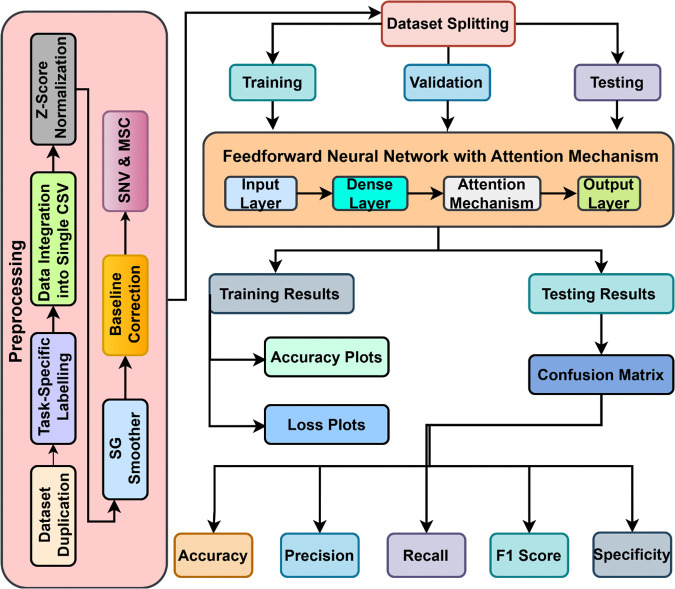
The block diagram of the proposed methodology utilized for the classification of three stage PET martials.

The integrated dataset was organized into a single CSV file containing essential columns: Wavelength, Absorbance, Reference Signal, Sample Signal, Variation, Sample num, Category, and Sensor id. In this structure, “Variation” indicates the specific classification task, “Sample num” provides a unique identifier for each spectral reading, “Category” represents the binary classification label, and “Sensor id” identifies the spectrometer that captured the data. This step was crucial in minimizing the variations in the scales of the features, so as to have all the features make an equal contribution to the model [37]. Furthermore, label encoding as well as one-hot encoding techniques were used to transform categorical variables into numerical form because deep learning models require the input data in some numerical form. The dataset was then split into training and testing sets using an 80:20 ratio to make sure to have enough data for using them for training and testing the system respectively. For classification, the proposed HAttFFNN model was used which extracts the detailed and minute features. These embeddings were then concatenated and again passed through fully connected layers before using SoftMax activation for classification [38]. TensorFlow was adopted for model implementation and the performance metrics included precision, recall, F1 score, accuracy and specificity. It has been conducted on the powerful computing machine with Intel Xeon CPU, Nvidia Tesla V100 GPU, 64GB RAM and 1TB SSD which allows for a high speed of convergence when training the model and processing a large amount of data. Fig 1 represents the proposed overall architecture used in the research.

### 3.1. Dataset

**Fig 2:**
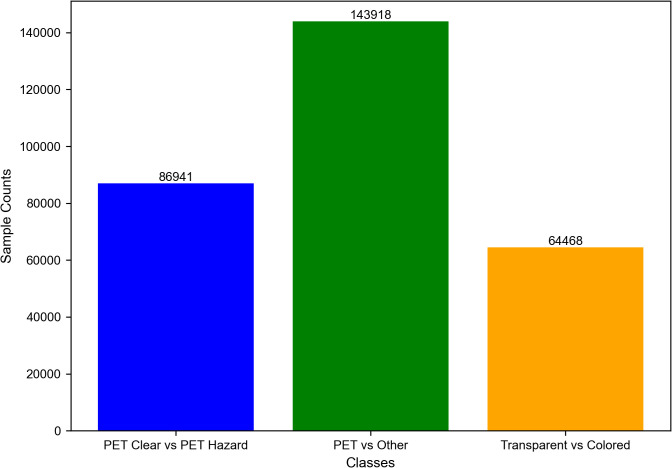
Dataset samples.

The dataset employed in this study consists of 295,327 samples that were obtained for the purpose of classification of different kinds of plastics based on their spectroscopic characteristics. The dataset includes spectral data which was collected in the near-infrared (NIR) range from the 900–1700 nm. Reflectance values have been recorded using the DLP NIR scan Nano Evaluation Module (EVM) and then subsequently preprocessed to enhance the spectral quality and to minimize the noise. To reduce the spectral noise, the Savitzky-Golay filter has been applied with a window size of 11 and a polynomial order of 2. Additionally, the baseline correction was performed to eliminate the background interferences. Standard Normal Variate (SNV) and Multiplicative Scatter Correction (MSC) techniques have been used to correct for scattering effects and standardize the spectral intensities. Feature extraction was carried out by identifying the absorbance peaks from the smoothed reflectance spectra. Peaks have been detected based on a prominence threshold of 0.005 and a minimum peak distance of 10 nm. These preprocessing steps basically ensures high-quality spectral data, facilitating the accurate classification of the plastic materials based on their optical properties. This dataset helps identify absorbance peaks for different plastic materials, distinguishing them based on optical properties. It contains several significant parameters including absorbance, wavelengths, reference signals, sample signals and category properties which are very important in achieving the proper data analysis and computation functions as well as in the actual classification of different types of plastics. The dataset is divided into three categories: Clear PET, Other PET, and Transparent Other plastics. In particular, the dataset is made up of the following samples of Clear, Hazard, Other PET with 86,941, 143918 and 64,468 respectively while Transparent Other has 143918. These categories are used for the binary classification tasks such as the classification of PET plastics from other types of plastics, differentiation between the transparent and colored type of plastics and in the classification of clear PET from contaminated PET. The large and highly diverse dataset provides great scope for different sets of plastic types can be used to create a proper baseline in training and testing of the novel HAttFFNN model. Due to the capability of the spectroscopic data for each sample, the dataset facilitates the feature extraction that improves the classification performance and the efficiency of the model for identifying plastics. Fig 2 represents the distribution of the samples in the dataset.

**Fig 3:**
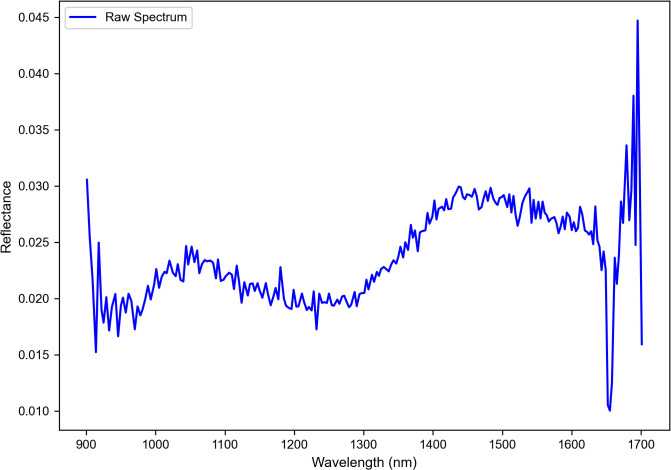
Raw spectra data.

Spectral data in the near-infrared (900–1700 nm) have been collected using the DLP NIR scan Nano Evaluation Module (EVM) and then preprocessed to enhance the quality by reducing the noise with a Savitzky-Golay filter with a window size of 11 and polynomial order of 2 and applying baseline correction. Standard Normal Variate and Multiplicative Scatter Correction have been used to correct the scattering effects and to also standardize the intensities. Key spectral features were extracted by identifying the absorbance peaks using the find_peaks function with a prominence threshold of 0.005 and a minimum peak distance is 10 nm, which highlights the characteristic absorption bands of the different plastic materials. Fig 3 here represents the raw spectral data.

**Fig 4:**
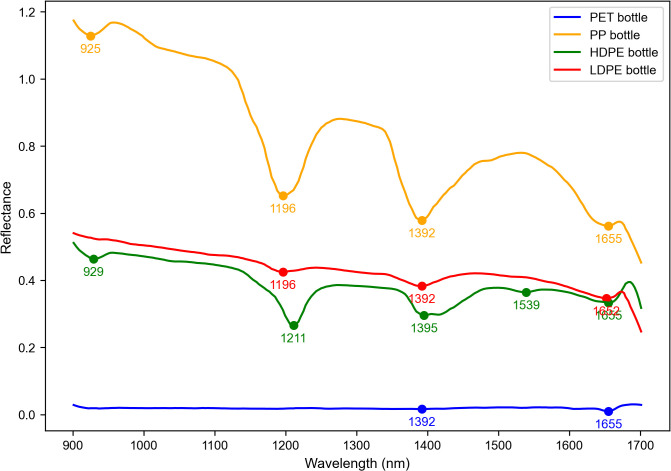
Mean NIR spectra of different plastic bottle materials.

Fig 4 here represents the mean NIR spectra (900–1700 nm) of PET, PP, HDPE, and LDPE plastic bottles, which are preprocessed using the Savitzky-Golay filtering (window size: 11, polynomial order: 2), SNV, and MSC for performing the noise reduction. Distinct absorption peaks basically differentiate the materials, with PET showing the characteristic absorption at 1392 nm and 1655 nm, while HDPE, PP, and LDPE exhibit their peaks at 925 nm, 1196 nm, 1211 nm, 1395 nm, and 1539 nm, corresponding to the C-H stretching and the bending vibrations. Absorbance at 1392 nm and 1655 nm has been selected as the key spectral features for classifying the PET versus other plastics in the deep learning model.

**Fig 5:**
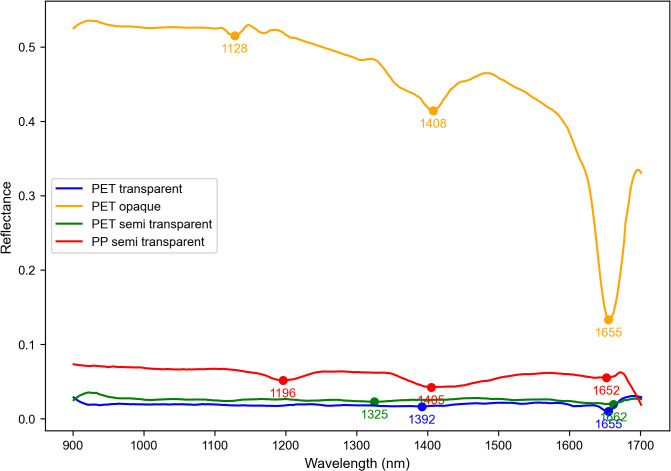
Mean NIR spectra of PET bottles with different transparency levels.

Fig 5 basically represents the mean NIR spectra of the PET bottles which is then categorized by transparency, transparent (blue), semi-transparent (green), and opaque (orange), along with the semi-transparent PP (red) for the comparison. Preprocessed using the consistent filtering techniques, the spectra reveal the key differences, transparent PET has very minimal variations with peaks at 1392 nm and 1652 nm, semi-transparent PET shows additional absorption at 1325 nm and 1405 nm due to the pigmentation, and opaque PET exhibits the strong absorption at 1128 nm, 1408 nm, and 1655 nm. These variations, influenced by light transmittance and pigment concentration, which serve as the critical features for the second-level classification task of distinguishing the transparent from the colored PET bottles in the deep learning model.

**Fig 6:**
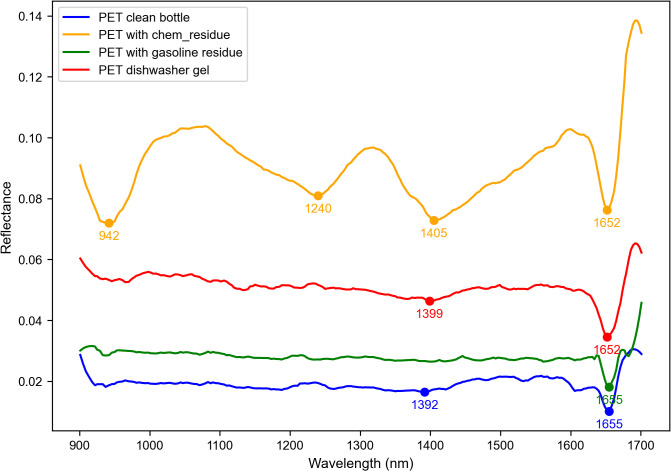
Mean NIR spectra of clean and contaminated PET bottles.

Fig 6 here represents the mean spectra of the clean and the contaminated PET bottles, which show how residue presence alters the spectral features. Clean PET (blue) exhibits the strong absorption at 1392 nm and 1655 nm, while contaminated PET spectra reveal the distinct changes, chemical residues (green) which introduce absorption at 1352 nm and 1652 nm, gasoline residues (yellow) which cause shifts at 942 nm, 1240 nm, and 1405 nm, and dishwasher gel (red) create a peak at 1399 nm. These variations basically result from the molecular interactions between the PET and the contaminants, affecting C-H stretching vibrations and the hydrogen bonding. This multi-tiered classification approach refines the PET identification, distinguishing it first from the other plastics, then by transparency, and finally by the contamination, optimizing the sorting accuracy for better recycling and sustainability.

### 3.2. Data preprocessing

The techniques used in data preprocessing important in this study are significant for converting the raw dataset into a format coherent with the requirements of the deep learning model assessment. The preprocessing step is a process that covers a number of tasks aimed at fulfilling various needs to impact the model’s performance as well as data credibility and handling efficiency for the learning algorithm.

***Savitzky-Golay Smoothing Filter:*** The Savitzky-Golay (SG) filter is basically a digital smoothing filter that fits a polynomial of a specified order to a set of data points within a moving window. This method basically preserves the spectral features while reducing the noise. The smoothing is achieved by convolving the signal with a set of the weighting coefficients that derives from the least-squares polynomial fitting. Smoothed signal $y_{i}^{SG}$ at each point *i* is computed as:


$$y_{i}^{SG}=\sum_{j=-m}^{m}c_{j}x_{i}+j$$ (1)

Where $y_{i}^{SG}$ is smoothed value at point i, $x_{i}+j$ are the original spectral data points within the window, $c_{j}$ are the convolution coefficients obtained from polynomial fitting, 2m+1 is the window size.

***Baseline Correction:*** Baseline correction is another preprocessing step in our methodology that removes low-frequency background signals and the systematic distortions which are caused by the instrumentation. One common approach is asymmetric least squares (ALS) smoothing, which basically estimates and subtracts the baseline from the spectrum. The baseline b(x) is basically obtained by solving:


$$\sum_{i=\text{1}}^{n}w_{i}{\left(y_{i}-b_{i}\right)}^{\text{2}}+\lambda \sum_{i=\text{2}}^{n}{\left(b_{i}-\text{2}b_{i-\text{1}}+b_{i-\text{2}}\right)}^{\text{2}}$$ (2)

where $y_{i}$ is the observed spectral intensity, $b_{i}$ is the estimated baseline, $w_{i}$ is weights that controls the asymmetry of the baseline fitting, and λ is the smoothing parameter that balances fidelity and smoothness. By iteratively adjusting the weights, the baseline is estimated and subtracted from the spectrum.

***Standard Normal Variate (SNV):*** SNV standardizes the spectral data by centering and scaling each spectrum independently:


$$x_{i}^{SNV}=\frac{x_{i}-\mu }{\sigma }$$ (3)

Where $x_{i}$ is the original spectral intensity at wavelength i, μ is the mean of the spectrum, σ is the standard deviation of the spectrum.

***Multiplicative Scatter Correction (MSC):*** MSC corrects for scattering variations by linearly transforming each spectrum based on a reference spectrum $x^{ref}$:


$$x_{i}^{MSC}=\frac{x_{i}-b}{a}$$ (4)

Where a and b are the regression coefficients obtained from:


$$x_{i}=ax_{i}^{ref}+b+\in_{i}$$ (5)

***Dataset duplication:*** In order to perform three types of binary classification, a full copy of the dataset is made and then divided into three independent segments each of which, represents a particular classification problem [39]. These tasks are as follows:

- **PET vs. Other Plastics:** The task is specifically on sorting the plastic samples into PET and all the other types of plastic. This categorization enables the model to differentiate between PET and other types of plastics used in several applications.
- **Transparent vs. Colored Plastics:** This task involves categorizing plastics in two categories. Depending on the optical properties of plastics, that is, whether it is clear or pigmented. This has implications forthe recycling processes as well as identification of the product.
- **PET Clear vs. PET Hazard:** This particular task is directed towards the PET plastics with the aim of sorting the PET plastics by level of contamination, unambiguous, or contaminated. According to this classification, it is possible to evaluate not only the quality of obtained PET plastics, but also their recyclability.

***Labeling:*** After the dataset is split into the required problem types, labeling is given to ensure that each sample is given a categorical label based on its features [40]. For each binary classification task, the following labels are used:

- **PET vs. Other:** In this task, samples are classified into PET and Other depending on the identity of the plastic material.
- **Transparent vs. Colored:** Samples are labeled as Transparent or Colored in relation to material’s look when it is illuminated with a light source.
- **PET Clear vs. PET Contaminated:** The samples in this task are classified as being from either the Clear PET (no other contents or impurities) or Contaminated PET (with other materials included).

***Data Integration:*** Once the dataset is split and pre-labeled, the data for all three classification tasks are merged into one CSV file. This integration step resolves complexity and makes it easier for the program to handle it with the aim of feeding it to the model for training [41]. The dataset contains the following essential columns:

- **Wavelength (nm):** The range of electromagnetic radiation that is employed in the determination of the absorbance of the material. The absorbance spectrum is measured across the wavelength for each of the samples that are taken.
- **Absorbance (AU):** The interaction of this material with light appears as an absorbance value for each specific wavelength.
- **Reference signal:** A signal, obtained during the measurement process, which is used for comparison with the sample signal.
- **Sample signal:** A signal reconstructed from the sample received signal, used as a comparison point with the reference signal to determine the characteristic of the material.
- **Variation:** This column defines the nature of classification exercise for the sample so as to distinguish between the three tasks.
- **Sample num:** A sample ID number that is assignable to every spectral reading in order to provide a unique serial number that will make it easier to track and relate a particular sample to the dataset.
•**Category:** The flag label given for each sample stating to which class the particular sample belongs to.
- **Sensor id:** This column specifies which particular spectrometer was applied to take the spectral data and gives certain information about the data collection work.

***Normalization:*** Normalization is one of the most important processes used in preprocessing. Basically, Standard Scaling (Z-score normalization) is applied to all finite or continuous variables in the dataset. Normalization on the other hand is a pre-processing technique whose aim is to align the attributes such that, on average they equal zero and they have variance equal to one [42]. This enables us to avoid situation whereby one main feature has a large scale or a large variance that tends to overpower the rest in the learning process. The formula for Z-score normalization is given as:


$$z=\frac{x-\mu }{\sigma }$$ (6)

where, x is the feature value, μ is the mean of the feature, and σ represents the standard deviation of the feature in the database. This normalization step is important for deep learning models to work the models converge during the training phase and to out-perform by making the model to focus on the relative importance of the features rather than the magnitude. The model is then freed from having to recognize the data in raw form, and this simplifies its task of making sense of it and making correct projections pertaining to it.

***Dataset Splitting:*** The data is then split into training and testing subsets when the dataset is preprocessed and normalized. This step serves to check the performance of the model on unseen data to prevent overfitting bythe model. It maintains the ratio of 80:20, where 80% of the data is used in training the model, while 20% is used for the model testing and validation. This split is useful to guarantee that the model has a sufficient number of examples to learn on, at the same time that a separate aggregate of data is available for model assessment [43]. The 80:20 split is a measure that is commonly used in machine learning to avoid the experiments results being skewed by the training data.

### 3.3. State-of-the-art models

- ***Long Short-Term Memory Networks (LSTM):*** Long Short-Term Memory (LSTM) network is an RNN that is specifically designed for addressing a long-term dependency problem. In contrast to traditional kinds of RNNs LSTMs make use of memory cells and gates that allow information to be retained or forgotten. In an LSTM, the basic three gates are input gate, the forget gate, and the output gate which all have their own significant role to perform [44]. The key equations for LSTM are:

$$f_{t}=\sigma \left(W_{f}\cdot \left[h_{t-\text{1}},x_{t}\right]+b_{f}\right)\;(\text{Forget Gate})$$ (7)

$$i_{t}=\sigma \left(W_{i}\cdot \left[h_{t-\text{1}},x_{t}\right]+b_{i}\right)\;(\text{Input Gate})$$ (8)

$${\tilde{C}}_{t}=tanh\left(W_{c}\cdot \left[h_{t-\text{1}},x_{t}\right]+b_{c}\right)\;(\text{Candidate Cell State})$$ (9)

$$C_{t}=f_{t}*C_{t-\text{1}}+i_{t}*{\tilde{C}}_{t}\;(\text{Cell State Update})$$ (10)

$$O_{t}=\sigma \left(W_{o}\cdot \left[h_{t-\text{1}},x_{t}\right]+b_{o}\right)\;(\text{Output Gate})$$ (11)

$$h_{t}=O_{t}*tanh\left(C_{t}\right)\;(\text{Final Output State})$$ (12)

Here, $f_{t}$, $i_{t}$, and $O_{t}$ are the forget, input and output gates, $C_{t}$ is the cell state, $h_{t}$ is the hidden state, $x_{t}$ is the input, and σ represents the sigmoid activation function. This model is used extensively for any task that has dependencies in sequence information such as time series prediction, speech recognition and natural language processing mainly because of its capability to handle the vanishing gradient problem and long-term memory.

- ***Gated Recurrent Unit (GRU):*** GRU is an advanced and structurally simpler version of the Recurrent Neural Network which is mainly developed to handle sequential data. LSTM networks have dependencies over long time sequences of data, while simpler to implement than the LSTM, just like the GRU. As a form of recurrent neural network, GRUs involves two gates they include the update gate and the reset gate [45]. The so- called update gate defines how much of the past information is preserved and the reset gate defines the strength of the previous state’s contribution to the current state. The key equations for GRU are:

$$z_{t}=\sigma \left(W_{z}\cdot \left[h_{t-\text{1}},x_{t}\right]+b_{z}\right)\;(\text{Update Gate})$$ (13)

$$r_{t}=\sigma \left(W_{r}\cdot \left[h_{t-\text{1}},x_{t}\right]+b_{r}\right)\;(\text{Reset Gate})$$ (14)

$${\tilde{h}}_{t}=tanh\left(W_{h}\cdot \left[h_{t-\text{1}},x_{t}\right]+b_{h}\right)\;(\text{Candidate State})$$ (15)

$$h_{t}=\left(\text{1}-z_{t}\right)*h_{t-\text{1}}+z_{t}*{\tilde{h}}_{t}\;(\text{Final Output State})$$ (16)

Here, $z_{t}$ and $r_{t}$ are the update and reset gates, ${\tilde{h}}_{t}$ is the candidate state, $h_{t}$ is the final output state, $x_{t}$ is the input, and σ represents the sigmoid activation function. By its nature, it is suitable for temporal data especially time-series or spectral data, because of the problem of vanishing gradients and in terms of computational efficiency.

- ***ResNet:*** ResNet is a revolutionary advancement in deep learning that solves the gradient vanishing problem in training extremely deep artificial neural networks. ResNet presents residual connections which practically enable the model to learn residual functions in place of direct mappings. These skip connections help to jump over one or more layers so that the gradients can pass right through the network to make optimization easier to accomplish [46]. The building block of ResNet can be expressed as:

$$y=F\left(x,\left\{W_{i}\right\}\right)+x$$ (17)

Here, x is the input, $F\left(x,\left\{W_{i}\right\}\right)$ is the residual function learned by the network, and y is the output. Identity mapping (x) helps the network to pass the information from the previous layers to the next layers, which makes training of deep networks easier. ResNet has shown excellent performance in image classification, but applying fundamental concepts of ResNet Research to other areas such as spectroscopic analysis is possible owing to its capability of feature inception.

- ***1D Convolutional Neural Network (1D-CNN):*** 1D Convolutional Neural Networks (1D-CNNs) are specific types of deep learning models or architectures particularly suitable for data in sequence such as time series signals and texts. Compared to the typical neural network architecture, 1D-CNNs convolve over the temporal dimension only so that, by doing so, they can learn spatial hierarchies over the input sequence [47]. A single convolutional layer in a 1D-CNN can be expressed as:

y=σ(W*x+b) (18)

Here, W represents the convolutional kernel, * denotes the convolution operation, *x* is the input sequence, *b* is the bias term, and σ is the sigmoid activation function. All these models can perform extra operations such as convolution, pooling in order to lower dimension and make features more resistant to errors. These models are extremely powerful in cases where the local dependencies in sequences are required such as in signal predicting, time series predictions or even opinion mining in text.

- ***Multi-Head Neural Network***: Multi-head Neural Networks (MHNNs) are a type of neural network architecture containing several parallel-moving processing units, which we call “heads” to jointly extract different representations of the data. This type of architecture is especially beneficial for datasets which have multiple patterns of different characteristics because each head can be trained on different aspects of input features. Each of the heads makes its own predictions and these are accumulated in order to make the final prediction so as to capture many different forms of feature interactions and dependencies in the data [48]. The main use, therefore, of MHNNs is that they offer a much more comprehensive description of the input by making use of different perspectives of the same data. The general form of the MHNN can be written as follows:

$$y=softmax\left(\sum_{i=\text{1}}^{N}{head}_{i}(x)\right)$$ (19)

where *y* is the output, *N* is the number of heads, x is the input, and ${head}_{i}(x)$ is the output from the $i^{th}$ head. Each head performs computations such as feature extraction and transformation independently, with the final decision being based on the combined outputs.

- ***VGG 16:*** VGG16 is a deep network CNN architecture, that is very simple, but very effective for many computer vision tasks. Originally, it was developed for image classification problems, but it has been modified for usage with structured data like CSVs by considering the input data to consist of a flattened vector. The model is constructed from the 16 layers, 13 of which are convolutional layers and 3 are fully connected layers. Every convolutional layer applies small kernels of 3 × 3, which allows them to obtain high accuracy of data features. For CSV data the input undergoes some preprocessing steps where the data is reshaped to conform to the required model and then the general VGG16 model architecture is employed [49]. The general architecture can be expressed as:

y=softmax(FC3(ReLU(FC2(ReLU(FC1(x)))))) (20)

where *x* represents the flattened input vector, FC1, FC2, and FC3 are the fully connected layers, and ReLU is the activation function used between the layers. The output *y* is the predicted class probability distribution, obtained after passing through the final softmax function.

### 3.4. Model design and description

**Fig 7:**
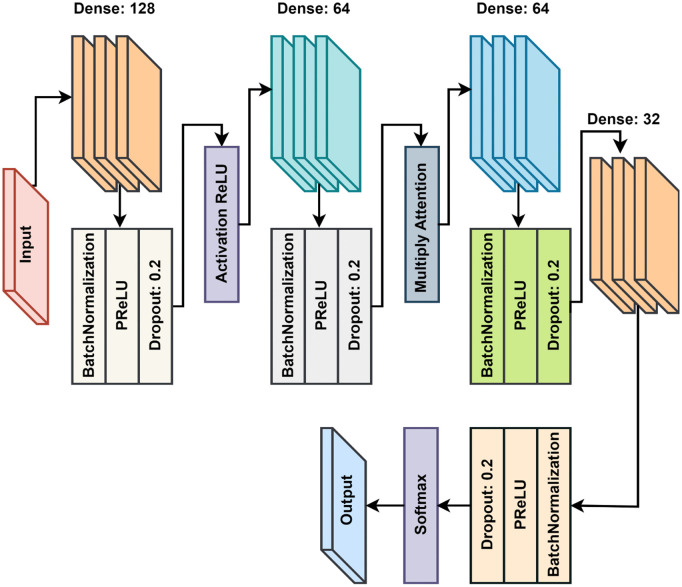
The architecture of the proposed HAttFFNN model.

The proposed model aimed at predicting the target variable into different categories through the results of the absorbance. It integrates both traditional dense layers and modern neural network techniques, such as residual connections, convolutional feature extraction, and attention mechanisms, to optimize performance. First, the data preprocessing step is essential, which means that the input data should be clean and aggregated consistently. The missing values for the selected input variables have been approximated to zero in order to ensure a strong structural integrity of the input dataset is retained. Nominal and ordinal data are converted into numerical form by one hot encoder technique and any type of ID variable such as sensor_id will be encoded. The target variable, Absorbance (AU), follows a skewed distribution that has been discretized into two classes by common convention. For binary classification tasks, these binarized labels are also one-hot encoded. We preprocess the input features in this case by scaling them using the StandardScaler so that all of them do not dominate the training process. Lastly, the dataset is divided into the training split of 80% and the validation split of 20% in order to train and assess a model. The proposed model combines the dense layers, residual connections, and the attention mechanism to achieve effective feature extraction and a classification task. The input layer takes and receives a feature vector of the specified shape. The first dense block uses a 128 filter dense layer with batch normalization, ReLU activation, and dropout to overcome vanishing gradients problems, and add a residual connection for steadier learning. In the second block the input is changed for one dimension convolution [50,51]. A dense layer with 64 filters and a kernel size of 3 extracts temporal or spatial dependencies and the subsequent Global Average Pooling layer dimensionality reduction. After the convolutional features, residual features are concatenated further for the feature representation [52]. It is the third dense block where an attention mechanism is applied to improve feature importance and thus, model interpretability and accuracy. When the feature representation is extracted, the attention weights, derived from a softmax activated dense layer, are applied on the feature representation. The fourth dense block continues feature extraction with the help of the dense layer with 32 units. The output layer uses a dense two-unit activation function with softmax activation to predict class probabilities. Fig 7 represents the architecture of the proposed HAttFFNN model used in our research. Table 2 represents the notations and definitions used in the algorithm. Although standard feed-forward neural networks are widely used in classification tasks, they often face challenges when it comes to dealing with the complex and noisy spectroscopic data due to their limited ability to focus on relevant features and interactions within the data. Our proposed hybrid attention mechanism-based feed-forward neural network (HAttFFNN) introduces a novel integration of hierarchical attention with the residual and convolutional layers, enabling the model to dynamically weigh and also emphasize the most informative spectral features while the mitigating noise and irrelevant information. This hybrid attention approach improves feature extraction beyond what traditional feed-forward networks or single-attention mechanisms can achieve, which results in enhancing the classification accuracy, robustness, and interpretability specifically tailored to the nuances of spectroscopic material classification. Proposed HAttFFNN model is summarized in Algorithm 1.

**Table 2:** Notations and definitions used in the algorithm.

Symbols	Description
D	Dataset containing features and target variable
X	Feature matrix extracted from the dataset
Y	Target variable representing absorbance value
T	Number of training epochs
B	Batch size used for training
α	Learning rate for the Adam optimizer
$\theta_{model}$	Model parameters to be optimized during
N	Total number of samples in the dataset
$X_{filled}$	Features after handling missing values
$\mu_{X},\sigma_{X}$	Mean and standard deviation of features X, used for normalization
$X_{scaled}$	Features after normalization
$Y_{binary}$	Binary version of labels, threshold by the median value
$Y_{encoded}$	One-hot encoded version of the binary labels
$R_{dense}$	Output of the dense layer in the residual connection
$H_{res}$	Output of the residual block after normalization and activation
$F_{conv}$	Features extracted using convolutional layers
$F_{conv\_avg}$	Global average-pooled features from convolutional layers
$H_{combined}$	Concatenated features from residual and convolutional layers
$W_{attention}$	Attention weights computed using a dense layer with softmax
$H_{attention}$	Features after applying attention mechanism
$X_{train\;}$	Training feature set
$y_{train}$	Training target variable
$X_{val\;}$	Validation feature set
$y_{val}$	Validation target variable
θ	Parameters (weights and biases) of the model
$L_{total}$	Total loss calculated during training
Dense	Fully connected layer in the neural network
ReLU	Rectified Linear Unit activation function
Dropout	Regularization technique to prevent overfitting
Adam	Optimizer algorithm used for updating the model parameters
Softmax	Activation function for converting outputs into probabilities
CM	Matrix showing the performance of classification predictions

**Algorithm 1:** Three stage martial classification using proposed HAttFFNN model

1: **Input:** $D,\;T,\;B,\;\alpha ,\;N,\theta_{model}\;$

2: **Preprocessing:**

2.1: Handling missing values:

2.2: $X_{filled}=fill\_na(X)$

2.3: Normalize features:

2.4: $X_{scaled}=\frac{X-\mu X}{\sigma X}$

2.5: Binary targets:

2.6: $Y_{binary}=binarize(Y,\;threshold=median(Y))$

2.7: One-hot encoded labels:

2.8: $Y_{encoded}=one\_hot(Y_{binary})$

3: **Dataset Split:** $Divide\;D\;into\;\left(X_{train},y_{train}\right)\;and\;\left(X_{val},y_{val}\right)$

4: **Model Initialization:**

4.1: Residual connection:

4.2: $R_{dense}=Dense(X,\theta_{res})$

4.3: $H_{res}=BatchNorm(ReLU(R_{dense}))$

4.4: Convolutional features:

4.5: $F_{conv}=Conv\text{1}D(expand\_dims(X))$

4.6: $F_{conv\_avg}=GlobalAvgPooling\text{1}D(F_{conv})$

4.7: Concatenation:

4.8: $H_{combined}=concat(H_{res},F_{conv\_avg})$

4.9: Attention mechanism:

4.10: $W_{attention}=Dense(H_{combined},\;softmax)$

4.11: $H_{attention}=H_{combined}\cdot W_{attention}$

4.12: Output:

4.13: $Y_{pred}=Dense(H_{attention},softmax)$

5: **Training Loop:**

5.1: For epoch=1 to T:

5.2: Split data into mini-batches:

5.3: $D_{batch}=\{(X_{batch},Y_{batch}\}$

5.4: For each batch:

5.5: Forward pass:

5.6: $Y_{batch}=Model(X_{batch},\theta_{model})$

5.7: Backward pass:

5.8: $\theta_{model}\leftarrow \theta_{model}-\alpha \cdot \nabla_{\theta_{model}}L_{batch}$

5.9: End for

5.10: End for

6: **Evaluation:**

6.1: Compute validation:

6.2: $Y_{val}=Model(X_{val},\theta_{model})$

6.3: Compute confusion matrix:

6.4: $CM=confusion\_matrix(Y_{val},argmax(Y_{val}))$

7: **Output:** $\theta_{model}$

### 3.5 Evaluation metrics

The performance of the proposed HAttFFNN model is therefore examined using a range of evaluation metrics, each of which quantifies the model’s performance in the context of different classification perspectives. These are precision, recall, F1 score, accuracy, specificity, and a confusion matrix pertinent to having overall features of the strengths and weaknesses of the model [53–57].


$$Precision=\;\frac{TP}{TP+FP}$$ (21)

$$Recall=\frac{TP}{TP+FN}$$ (22)

$$F\text{1}\;Score=\text{2}\times \frac{Precision\times Recall}{Precision+Recall}$$ (23)

$$Accuracy=\frac{TP+TN}{TP+TN+FP+FN}$$ (24)

$$Specificity=\frac{TN}{TN+FP}$$ (25)

***Confusion Matrix:*** The confusion matrix also gives the exact number of true positive, true negative, false positive, and false negatives that the model has identified. It gives information on the sort of mistakes that the model makes as well as promptly recognize repetitive misclassification [58]. This can be represented graphically and yet another conventional visualization technique that can be used is the heatmap.

***T-Test:*** The paired t-test was also used to statistically determine the mean performance of the proposed model with each of the baseline models. The test assesses that the means of the differences of paired results are not significantly equal to zero. In our case, it can be used to establish whether the increase in accuracy of the proposed model is significant. When the p-value is less than 0.05, it indicates that there is a significant difference [59]. Mathematically, it can be represented as:


$$t=\frac{\overset{―}{d}}{s_{d}/\sqrt{n}}$$ (26)

Where:

- $\overset{―}{d}$ is the mean of the differences between paired observations
- $s_{d}$ is the standard deviation of the differences
- $\sqrt{n}$ is the number of pairs

***Wilcoxon Signed-Rank Test*** The Wilcoxon signed-rank test was applied as a non-parametric equivalent to the t-test to prove statistical significance without requiring the distribution to be normal. It compares the differences between two items and ranks the differences between pairs [59]. In this paper, it has been ascertained that the proposed model is superior across various other proposed models. The p-value of less than 0.05 indicates that there is a statistically significant difference.


$$W=\sum_{i=\text{1}}^{n}R_{i}$$ (27)

Where:

- $R_{i}$ is the rank of the absolute difference between the $i^{th}$ pair
- The sum is calculated over positive or negative ranks, whichever is smaller

## 4. Experimental result and discussion

This research uses a dataset of 295,327 samples that consists only of samples specifically chosen for the classification of different types of plastics depending on the spectroscopic profile. The set of parameters presented includes absorbance, wavelengths, reference signals, sample signals, and categorical attributes in order to provide the possibility of comprehensive data analysis and classification. The dataset is categorized into three primary classes, i.e., Clear PET, PET other, and transparent colored plastics consisting of 86,941, 143,918 and 64,468 samples respectively. These categories allow for performing binary and multi-class classification tasks, including PET plastics from other types, transparent plastics from colored ones, and clean clear PET from contaminated samples. For model training and validation, the dataset was split into training and validation sets using an 80:20 ratio. The chosen dataset is highly variable and contains intricate spectroscopic features that augment the HAttFFNN model’s capacity to capture essential patterns and generalize correctly, making it appropriate to develop a strong framework for plastic type classification. It has been conducted on the powerful computing machine with Intel Xeon CPU, Nvidia Tesla V100 GPU, 64GB RAM and 1TB SSD which allows for a high speed of convergence when training the model and processing a large amount of data. The implementation was done through the TensorFlow 2.0 environment with the Keras interface. In this work, feature normalization and label encoding processes were done with Python programming language v 3.8, NumPy, pandas, and scikit-learn were used. The multi-head neural network architecture contained several dense layers each designed to learn different types of features of the spectroscopic dataset of the chosen chemical substances. The model used has been optimized using the Adam optimizer with an initial learning rate of 0.0001, while categorical cross-entropy has been used as the criterion for loss. Training was done over 100 epochs using a batch size of 16 while balancing the iterations for computational effectiveness as well as the model convergence.

### 4.1. Training and evaluation

**Fig 8:**
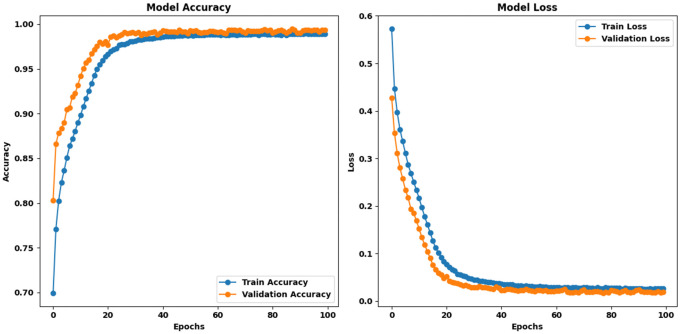
Training curves of proposed HAttFFNN model for Stage 1: PET Clear Vs PET Hazard.

**Fig 9:**
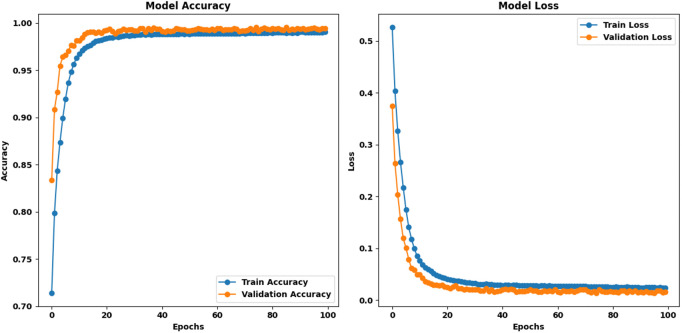
Training curves of HAttFFNN model for Stage 2: PET Vs Others.

**Fig 10:**
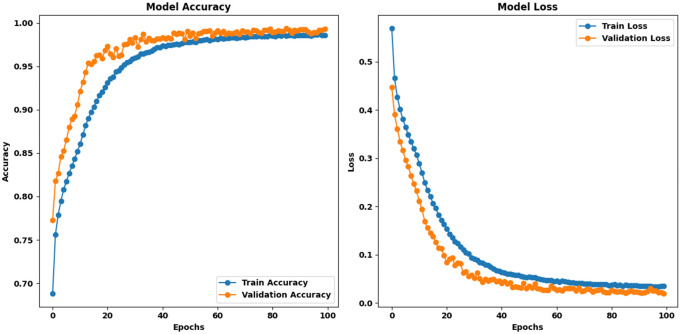
Training curves of proposed HAttFFNN model for stage 3: PET Coloured Vs PET Transparent.

- Proposed HAttFFNN model for Stage 1: PET Clear vs PET Hazard: When the proposed model was trained on the Stage 1: PET Clear vs PET Hazard dataset for 100 epochs, it outperformed all other state-of-the-art models considered in this study. The initial training accuracy was 64.93%, and the validation accuracy was 80.30%, with corresponding training and validation losses of 69.26 and 42.75, respectively. The model showed that there were no fluctuations in our training and validation curves and how the curves transitioned from one epoch to another. Finally, the model attained a complete training accuracy of 98.93%, a concurrent validation accuracy of 99.32%, complete training loss of 2.60, and validation loss 1.98. These results establish the proposed model as the best-performing one for this stage. Fig 8 represents the training curves of proposed HAttFFNN model for stage 1.

- Proposed HAttFFNN Model for Stage 2: PET vs Others: For the Stage 2: PET vs others dataset, the proposed model began with an initial training accuracy of 66.19% and a validation accuracy of 83.37%, accompanied by training and validation losses of 59.16 and 37.42, respectively. Moving forward, the curves of both the metrics did not show any oscillation and the smoothness was maintained throughout the training. After the 100 epochs, the model’s training accuracy was approximately 99.03% with approximately 99.42% of the validation accuracy and the final training loss was found to be 2.38 while the final validation loss was found to be 1.56. These data point to a consistency between epochs that holds true for this stage in the model as well. Fig 9 represents the training curves of proposed HAttFFNN model for stage 2.

- **Proposed HAttFFNN model for stage 3: PET Coloured vs PET Transparent:** On the Stage 3: PET Coloured vs PET Transparent dataset, the proposed model initially recorded a training accuracy of 63.02% and a validation accuracy of 77.30%, with training and validation losses of 65.69 and 44.71, respectively. Similar to the previous stages, both training and validation curves displayed smooth transitions without any fluctuations. At final epoch, the model reached a training accuracy of 98.57%, a validation accuracy of 99.28%, a training loss of 3.61, and a validation loss of 1.97. These results confirm that the proposed model is the best-performing model across all stages. Fig 10 represents the training curves of proposed HAttFFNN model for stage 3.

The training comparison among current models indicates that the proposed system delivers superior outcomes simultaneously for all three classification operations. The proposed model reaches the maximum validation accuracy through its ability to identify PET Clear vs. PET Hazard at 99.32% and PET vs. Others at 99.42% and PET Coloured vs. PET Transparent at 99.28%. The proposed model offers the best generalization capabilities because it reaches the lowest validation loss. Stable performance fluctuations distinguish the proposed model from other models because it produces consistent learning characteristics. The proposed model demonstrates its role as the most dependable and efficient approach for PET classification based on these research findings. Table 3 represents the training comparison of the proposed model with SOTA methods. The comparative results presented in the Table 3 and Table 7 are derived from our own experiments, where all the State-Of-The-Art (SOTA) models, including MHNN, GRU, VGG16, 1D CNN, LSTM, ResNet, and the proposed HAttFFNN, were trained and evaluated under consistent conditions using the same dataset and preprocessing pipeline. This ensures a fair and direct performance comparison of these architectures for the spectroscopic classification tasks addressed in this study.

**Table 3:** Comparative training analysis of the proposed model along with state-of-the-art methods.

Model	Stage	Training Accuracy	Validation Accuracy	Training Loss	Validation Loss	Training Curve Behavior	Validation Curve Behavior
	PET Clear Vs PET Hazard	79.23	84.88	0.046	0.034	Unstable Fluctuations	Unstable Fluctuations
**MHNN**	PET Vs Others	88.94	90.10	0.032	0.025	Unstable Fluctuations	Unstable Fluctuations
	PET Coloured Vs PET Transparent	87.62	91.62	0.036	0.022	Unstable Fluctuations	Unstable Fluctuations
	PET Clear Vs PET Hazard	87.08	97.43	0.026	0.070	Unstable Fluctuations	Unstable Fluctuations
**GRU**	PET Vs Others	89.26	96.28	0.021	0.830	Unstable Fluctuations	Unstable Fluctuations
	PET Coloured Vs PET Transparent	83.79	95.88	0.032	0.097	Unstable Fluctuations	Unstable Fluctuations
	PET Clear Vs PET Hazard	83.78	89.72	0.035	0.021	Unstable Fluctuations	Unstable Fluctuations
**VGG16**	PET Vs Others	89.99	93.16	0.025	0.015	Unstable Fluctuations	Unstable Fluctuations
	PET Coloured Vs PET Transparent	91.21	92.08	0.024	0.018	Unstable Fluctuations	Unstable Fluctuations
	PET Clear Vs PET Hazard	63.91	78.24	0.063	0.045	Unstable Fluctuations	Unstable Fluctuations
**1D CNN**	PET Vs Others	64.82	86.35	0.059	0.031	Unstable Fluctuations	Unstable Fluctuations
	PET Coloured Vs PET Transparent	66.86	79.43	0.058	0.041	Unstable Fluctuations	Unstable Fluctuations
	PET Clear Vs PET Hazard	83.68	90.13	0.038	0.019	Unstable Fluctuations	Unstable Fluctuations
**LSTM**	PET Vs Others	90.16	92.29	0.027	0.015	Unstable Fluctuations	Unstable Fluctuations
	PET Coloured Vs PET Transparent	90.55	91.48	0.029	0.019	Unstable Fluctuations	Unstable Fluctuations
	PET Clear Vs PET Hazard	81.36	87.14	0.040	0.028	Unstable Fluctuations	Unstable Fluctuations
**ResNet**	PET Vs Others	89.83	90.49	0.028	0.022	Unstable Fluctuations	Unstable Fluctuations
	PET Coloured Vs PET Transparent	90.43	91.90	0.025	0.019	Unstable Fluctuations	Unstable Fluctuations
	PET Clear Vs PET Hazard	**98.93**	**99.32**	**0.020**	**0.019**	**Stable Fluctuations**	**Stable Fluctuations**
**Proposed**	PET Vs Others	**99.03**	**99.42**	**0.023**	**0.015**	**Stable Fluctuations**	**Stable Fluctuations**
	PET Coloured Vs PET Transparent	**98.57**	**99.28**	**0.036**	**0.019**	**Stable Fluctuations**	**Stable Fluctuations**

### 4.2. Testing result

**Fig 11:**
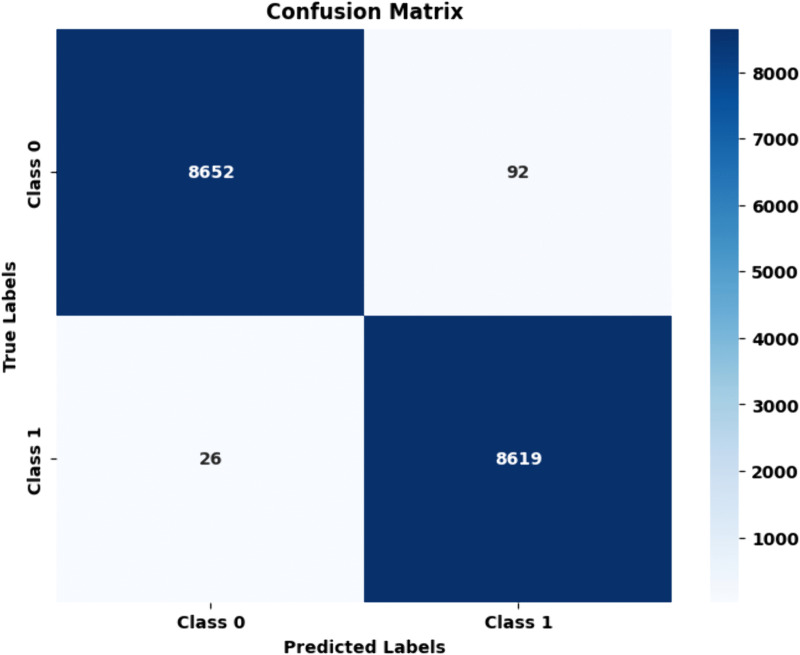
Confusion matrix of proposed HAttFFNN model for Stage 1: PET Clear Vs PET Hazard.

- Proposed HAttFFNN model for Stage 1: PET Clear vs PET Hazard: In comparison PET Clear vs PET Hazard, we see that the model achieves total correct classifications 8652 and 8619 for PET Clear and PET Hazard respectively with total misclassifications 92 instances from the PET Clear and 26 from PET Hazard classes. This made the accuracy achieved for both classes to be 99.33%. The precision values were 99.50% for PET Clear and 98.84% for PET Hazard and recall values were 98.95% and 99.97% for PET Clear and PET Hazard respectively. The F1 scores were 0.9923 of the PET Clear models and 0.9910 of the PET Hazard models with specificity of 0.9969 and 0.9970 respectively. These results suggest that the proposed model achieved far better classification accuracy over other SOTA models and was more consistent as well. Fig 11 and Table 4 confusion matrix and performance metric of the proposed HAttFFNN model for stage 1.

**Table 4:** Performance metric of the proposed HAttFFNN for stage 1: PET Clear vs PET Hazard.

Metric	PET Clear	PET Hazard
Accuracy	99.33	99.33
Precision	99.50	98.84
Recall	98.95	99.97
F1 Score	99.23	99.10
Specificity	99.69	99.70

**Fig 12:**
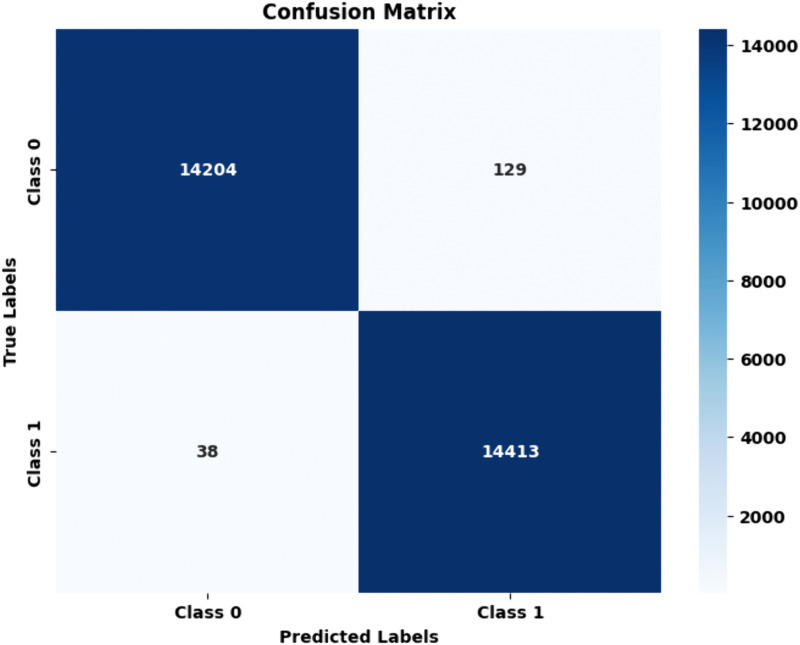
Confusion matrix of proposed HAttFFNN model for stage 2: PET Vs Others.

- Proposed HAttFFNN for stage 2: PET vs Others: Compared between PET and Others, the model correctly classified 14,204 of PET and 14,413 of Others and misclassified 129 PET and 38 Others. Both classes hit a 99.32% accuracy on test data. PET had a precision of 99.67% and a recall of 99.02%, while Others had a precision of 98.92% and a recall rate of 99.59%. The F1 scores increased to 99.34% for PET and 99.25% for Others while the specificity scores are at 99.69% and 99.73%. The fine-tuning of the proposed model offers SOTA performance than other models showing reliability and stability of the proposed method. Fig 12 and Table 5 confusion matrix and performance metric of the proposed HAttFFNN model for stage 2.

**Table 5:** Performance metric of proposed HAttFFNN model for stage 2: PET vs Others.

Metric	PET	Others
Accuracy	99.32	99.32
Precision	99.67	98.92
Recall	99.02	99.59
F1 Score	99.34	99.25
Specificity	99.69	99.73

**Fig 13:**
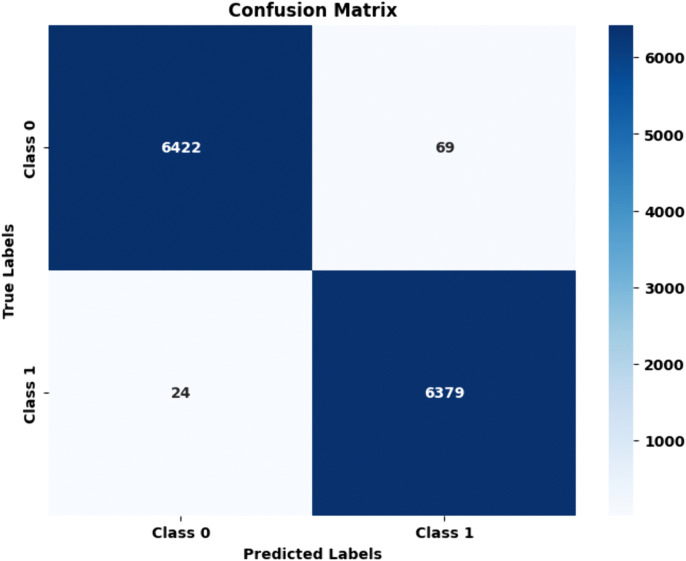
Confusion matrix of proposed HAttFFNN model for stage 3: PET Coloured Vs PET Transparent.

- Proposed HAttFFNN model for stage 3: PET Coloured vs PET Transparent: The proposed model classified 6422 samples on PET Coloured and 6379 on PET Transparent with a misclassification of 69 and 24 respectively. The total accuracy for both the classes were 99.28%. The values of Precision of PET Coloured were 98.87% and PET Transparent was 99.53% and the values of recall of PET Coloured being 99.53% and PET Transparent 98.84%. The values for F1 score of PET Coloured and PET Transparent were 99.20% and 99.18% and Specificity scores of PET Coloured and PET Transparent were 98.93% and 99.53%. The proposed model also outperformed other SOTA models in this classification task, providing the highest levels of accuracy and reliability. Fig 13 and Table 6 confusion matrix and performance metric of the proposed HAttFFNN for stage 3.

**Table 6:** Performance metric of proposed HAttFFNN model for stage 3: PET Coloured vs PET Transparent.

Metric	PET Coloured	PET Transparent
Accuracy	99.28	99.28
Precision	98.87	99.53
Recall	99.53	98.84
F1 Score	99.20	99.18
Specificity	98.93	99.53

**Table 7:** Comparative testing analysis of the proposed model along with state-of-the-art methods.

Model	Stage	Accuracy	Precision	Recall	F1 Score	Specificity
	PET Clear Vs PET Hazard	98.05	98.94/ 95.17	98.78/ 95.70	98.86/ 95.43	95.73/ 98.77
**MHNN**	PET Vs Others	98.85	99.67/ 91.97	98.94/ 97.05	99.30/ 94.36	97.05/ 99.04
	PET Coloured Vs PET Transparent	97.40	89.05/ 98.12	83.05/ 98.73	85.84/ 98.42	99.82/ 83.05
	PET Clear Vs PET Hazard	99.69	96.03/ 95.63	96.52/ 96.25	95.82/ 95.94	96.25/ 95.63
**GRU**	PET Vs Others	99.33	99.21/ 98.39	98.37/ 99.38	98.79/ 98.84	99.42/ 98.57
	PET Coloured Vs PET Transparent	98.64	99.13/ 98.07	98.05/ 99.19	98.58/ 98.63	98.05/ 97.95
	PET Clear Vs PET Hazard	99.16	99.32/ 98.36	99.55/ 97.17	99.44/ 97.76	99.17/ 99.30
**VGG16**	PET Vs Others	99.55	99.72/ 96.25	99.56/ 97.43	99.64/ 96.83	97.43/ 99.72
	PET Coloured Vs PET Transparent	97.65	95.77/ 98.56	86.83/ 99.59	90.89/ 99.07	98.62/ 86.83
	PET Clear Vs PET Hazard	99.23	98.70/ 93.39	93.07/ 98.76	95.84/ 95.96	98.76/ 93.13
**1D CNN**	PET Vs Others	98.61	96.99/ 98.65	98.66/ 96.96	97.82/ 97.80	96.96/ 98.66
	PET Coloured Vs PET Transparent	96.23	95.05/ 97.06	97.08/ 95.01	96.06/ 96.0	95.01/ 97.08
	PET Clear Vs PET Hazard	98.35	98.79/ 96.23	99.17/ 95.53	98.98/ 95.88	95.45/ 98.89
**LSTM**	PET Vs Others	99.49	99.60/ 97.45	99.67/ 97.45	99.63/ 97.45	97.45/ 99.72
	PET Coloured Vs PET Transparent	97.37	90.15/ 98.72	89.15/ 98.74	89.65/ 98.73	98.85/ 89.1
	PET Clear Vs PET Hazard	97.14	97.45/ 95.47	98.95/ 90.73	98.19/ 93.05	90.56/ 97.96
**ResNet**	PET Vs Others	98.32	99.35/ 94.03	99.29/ 95.37	99.32/ 94.69	95.47/ 99.45
	PET Coloured Vs PET Transparent	98.57	96.45/ 98.67	87.64/ 99.66	91.79/ 99.16	98.73/ 87.74
	PET Clear Vs PET Hazard	**99.33**	**99.50/ 98.84**	**98.95/ 99.97**	**99.23/ 99.10**	**99.69/ 99.70**
**Proposed**	PET Vs Others	**99.32**	**99.67/ 98.92**	**99.02/ 99.59**	**99.34/ 99.25**	**99.69/ 99.73**
	PET Coloured Vs PET Transparent	**99.28**	**98.87/ 99.53**	**99.53/ 98.84**	**99.20/ 99.18**	**98.93/ 99.53**

A comparative testing analysis shows that the proposed model provides excellent performance by achieving high levels of accuracy on every classification task. For PET vs. Others, the proposed model attains an accuracy of 99.32%, with precision, recall, and F1 scores surpassing 98.9%, ensuring high classification reliability. Similarly, for PET Coloured vs. PET Transparent, the model achieves 99.28% accuracy, maintaining balanced precision and recall values. Unlike other models, which show variations in precision-recall balance, the proposed model consistently delivers high scores across both classes in each task. Its strong generalization capability, as reflected in the near-perfect specificity values, establishes it as the most reliable model for PET classification. Table 7 represents the testing comparison of the proposed model with SOTA methods. Additionally, in S1 Appendix, we provide extended comparative analysis, including complete confusion matrices, training and validation curves, and detailed SOTA model performance results corresponding to Tables 3 and 7.

The comparison Table 8 of the proposed model and six other existing deep learning models (MHNN, GRU, VGG16, 1D CNN, LSTM, and ResNet) shows that the proposed model has better performance in terms of mean accuracy of 99.53% which remains higher than the other existing models. Test results included paired t-tests and Wilcoxon signed rank tests to confirm the significance between the performance difference. The findings conclude that all of the comparisons indicated statistically significant (p < 0.05) differences in favor of the proposed model. The proposed model was significantly superior to MHNN, 1D CNN, and ResNet, and in both tests the p-value is 0.000000 which is strong evidence against the null hypothesis. They showed that even in comparison to high-performance models, such as GRU or VGG16, the proposed model has kept the statistical advantage with t-test p-values, and p-values of 0.023957 and 0.000687 respectively. These results were further confirmed by Wilcoxon tests with all p-values coming up less than 0.05 confirming the reliability, strength, and improved general classification abilities of the proposed model on the various spectral classification scenarios.

**Table 8:** Statistical comparison of accuracy between the proposed model and existing model using T-Trest and Wilcoxon Signed-Rank Test.

Model	Proposed Mean	Model Mean	T-Statistics	T-Test P-Value	T-Test Significant (p < 0.05)	Wilcoxon Statistics	Wilcoxon P-Value	Wilcoxon Significant (p < 0.05)
**MHNN**	99.534031	97.704671	11.065424	0.000000	True	0.000000	0.001953	True
**GRU**	99.534031	99.109078	2.468765	0.023957	True	8.000000	0.048828	True
**VGG16**	99.534031	98.624947	4.245202	0.000687	True	2.000000	0.005859	True
**1D CNN**	99.534031	97.893589	8.983385	0.000000	True	0.000000	0.001953	True
**LSTM**	99.534031	98.499721	6.475634	0.000004	True	0.000000	0.001953	True
**ResNet**	99.534031	98.002472	8.502768	0.000000	True	0.000000	0.001953	True

The proposed model has indicated the superiority over the State-Of-The-Art (SOTA) models in the classification of PET materials at the various stages presented by its improved time complexity and the commendable runtime measures. At the stage, “PET Clear vs PET Hazard”, the proposed model achieves a time of 611 seconds which is better than ResNet (783 seconds), MHNN (745 seconds), GRU (2095 seconds), VGG16 (808 seconds), 1D CNN (806 seconds), and LSTM (2108 seconds). In the “PET vs Others” stage, the proposed model’s running time is 1207 seconds as compared to MHNN (1234 seconds), GRU (3754 seconds), 1D CNN (1330 seconds), LSTM (3652 seconds), and ResNet (1392 seconds), VGG16 (1224 seconds). For the “PET Coloured vs PET Transparent” stage, the proposed model has achieved the lowest run time of 514 seconds which is far ahead of MHNN (612 seconds), GRU (1707 seconds), VGG16 (610 seconds), 1D CNN (606 seconds), LSTM (1706 seconds), and ResNet (611 seconds). This uniform downward trend towards the run time on all the stages demonstrates that the proposed model implements the most efficient architectural elements and feature extraction strategies that allow the process to speed up the process without losing the potential of the classification improvement. These quantitative advances in time consumption indicate that the suggested model is a high-performance and competitive alternative to PET material classification activity compared with MHNN, GRU, VGG16, 1D CNN, LSTM, and ResNet. Table 9 represents the comparative analysis of model execution time across all classification stages.

**Table 9:** Time complexity comparison of proposed model with state-of-the-art methods across all classification stages.

Model	Stage	Time Taken
	PET Clear Vs PET Hazard	745 seconds
**MHNN**	PET Vs Others	1234 seconds
	PET Coloured Vs PET Transparent	612 seconds
	PET Clear Vs PET Hazard	2095 seconds
**GRU**	PET Vs Others	3754 seconds
	PET Coloured Vs PET Transparent	1707 seconds
	PET Clear Vs PET Hazard	808 seconds
**VGG16**	PET Vs Others	1224 seconds
	PET Coloured Vs PET Transparent	610 seconds
	PET Clear Vs PET Hazard	806 seconds
**1D CNN**	PET Vs Others	1330 seconds
	PET Coloured Vs PET Transparent	606 seconds
	PET Clear Vs PET Hazard	2108 seconds
**LSTM**	PET Vs Others	3652 seconds
	PET Coloured Vs PET Transparent	1706 seconds
	PET Clear Vs PET Hazard	783 seconds
**ResNet**	PET Vs Others	1392 seconds
	PET Coloured Vs PET Transparent	611 seconds
	PET Clear Vs PET Hazard	**611 seconds**
**Proposed**	PET Vs Others	**1207 seconds**
	PET Coloured Vs PET Transparent	**514 seconds**

## 5. Conclusion

This study provides an in-depth analysis of material classification using spectroscopic data by implementing and comparing a variety of state-of-the-art deep learning models, including LSTM, GRU, ResNet, 1D CNN, VGG16, and a novel proposed model, HAttFFNN. The spectroscopic data in the 900–1700 nm range were acquired using the DLP NIR scan Nano Evaluation Module (EVM) and underwent rigorous preprocessing to enhance signal quality. For noise reduction the Savitzky-Golay filter with a window size of 11 and polynomial order of 2 was applied. In addition, baseline correction, Standard Normal Variate (SNV), and Multiplicative Scatter Correction (MSC) were used to minimize background interference and scattering effects, ensuring robust input for classification. The proposed HAttFFNN model significantly outperformed all other deep learning approaches across all three classification stages. For Stage 1: PET Clear vs PET Hazard, the proposed HAttFFNN model achieved an accuracy of 99.33% for both PET Clear and PET Hazard, with precision scores of 99.50% and 98.84%, respectively. The recall rates were 98.95% for PET Clear and 99.97% for PET Hazard, along with F1 scores of 99.23% and 99.10%, and specificity values of 99.69% and 99.70%. These results demonstrate its superior performance compared to other models like VGG16, ResNet, and 1D CNN. For Stage 2: PET vs Others, the model achieved an accuracy of 99.32% for both PET and Others. The precision was 99.67% for PET and 98.92% for Others, while recall values were 99.02% for PET and 99.59% for Others. The F1 scores were 99.34% for PET and 99.25% for Others, with specificity scores of 99.69% and 99.73%. These Figures further confirm the novel model’s advantage over the other SOTA models such as LSTM, GRU, and ResNet. For Stage 3: PET Coloured vs PET Transparent, the HAttFFNN model achieved an accuracy of 99.28% for both PET Coloured and PET Transparent. Precision values were at 98.87% and 99.53% for PET Coloured and PET Transparent respectively while the recall rates were 99.53% for PET Coloured and 98.84% for PET Transparent. PET Coloured achieved a F1 of 99.20% and specificity of 98.93%, while PET Transparent had F1 of 99.18% and specificity of 99.53%. These results show higher performance of the proposed new model than the other existing models like 1D CNN, ResNet and multi-head neural networks. The core contribution of this work lies in the design and successful deployment of the HAttFFNN model, which combines hierarchical attention mechanisms and feed-forward neural networks to enhance feature extraction and classification reliability in complicated spectroscopic data. Despite achieving the optimal results, this work critically acknowledges limitations of both the traditional and the existing deep learning methods, particularly their difficulty in capturing subtle spectral variations and also their sensitivity to noise and scattering in real-world spectroscopic data. Traditional models lack the hierarchical feature extraction or adaptive attention mechanisms, which are essential for high-precision classification across nuanced material types. Although the proposed HAttFFNN model demonstrates high accuracy and robustness on the current dataset, its real-world applicability still requires careful consideration. Challenges such as the variability in spectroscopic data from several instruments, and the risks of overfitting due to dataset characteristics, and limited interpretability may affect its deployment in the industrial settings. To ensure the generalizability, future work will explore diverse datasets, apply regularization techniques, and will adopt explainable AI methods to improve both model reliability and the user trust in practical applications. In addition to its better performance compared to current models, the proposed method also turned out to be highly reliable on various stages of classification and hence a versatile solution in various application scenarios. In order to increase model transparency and reliability, future research will focus on real-time deployment strategies, edge computing platform integration, and interpretability techniques. Furthermore, the methodology will be extended to include other types of plastic materials and spectroscopic modalities to broaden its scientific and industrial impact. These initiatives will improve the model’s scalability and help to create more efficient, automated, and intelligent systems for material classification using spectroscopy.

## Supporting information

S1 Appendix This file contains all supplementary materials supporting the main manuscript, including full experimental results for all evaluated SOTA models. (PDF)
