# Supplementary material for: HAttFFNN: Hybridized attention mechanism-based feedforward neural network deep learning model for the plastic material classification of three stage materials on spectroscopic data
Source: PLoS One. 2025 Dec 2;20(12):e0336927. doi: 10.1371/journal.pone.0336927 (PMC12671785; doi:10.1371/journal.pone.0336927)
Supplement: S1 Appendix — (PDF) [file pone.0336927.s001.pdf]

S1 Appendix

Training and evaluation

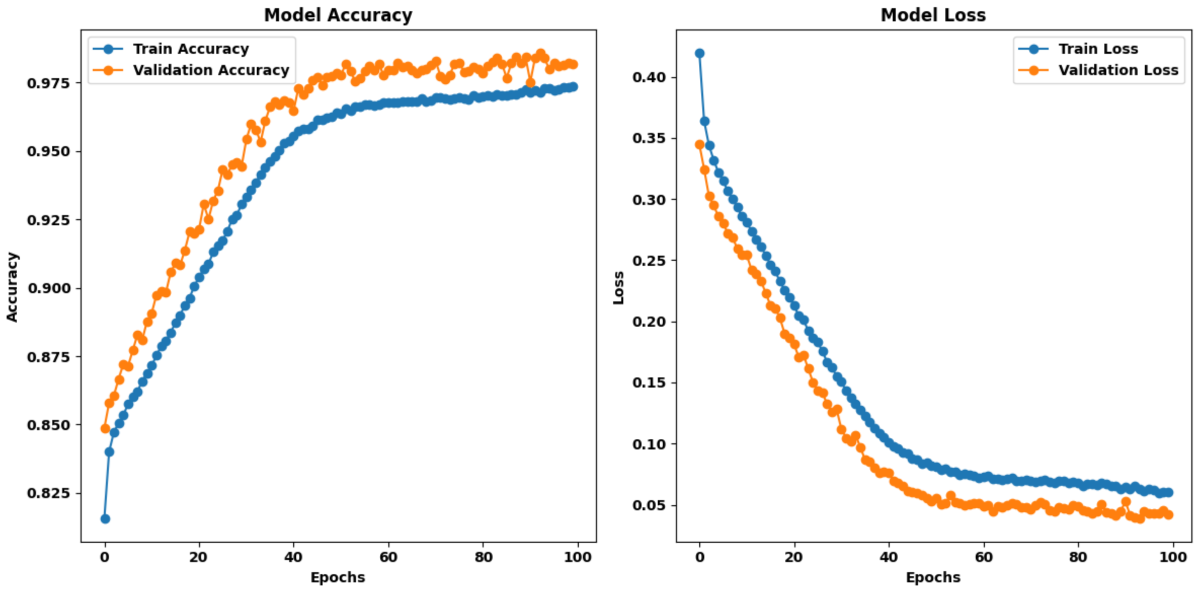

S1 Fig. A. Training curves of multi-head neural network (Stage 1: PET Clear Vs PET Hazard). During the training of the multi-head neural network on the Stage 1: PET Clear vs PET Hazard dataset for 100 epochs, the model obtained the training accuracy of 79.23% and the validation accuracy of 84.88%. The initial training loss was 46.99, whereas the validation loss was 34.45. In each training session, the training curve did not experience dramatic changes, and gradually flattened as the process continued. However, a validation curve showed more fluctuations and this represent variability in the performance during evaluation.

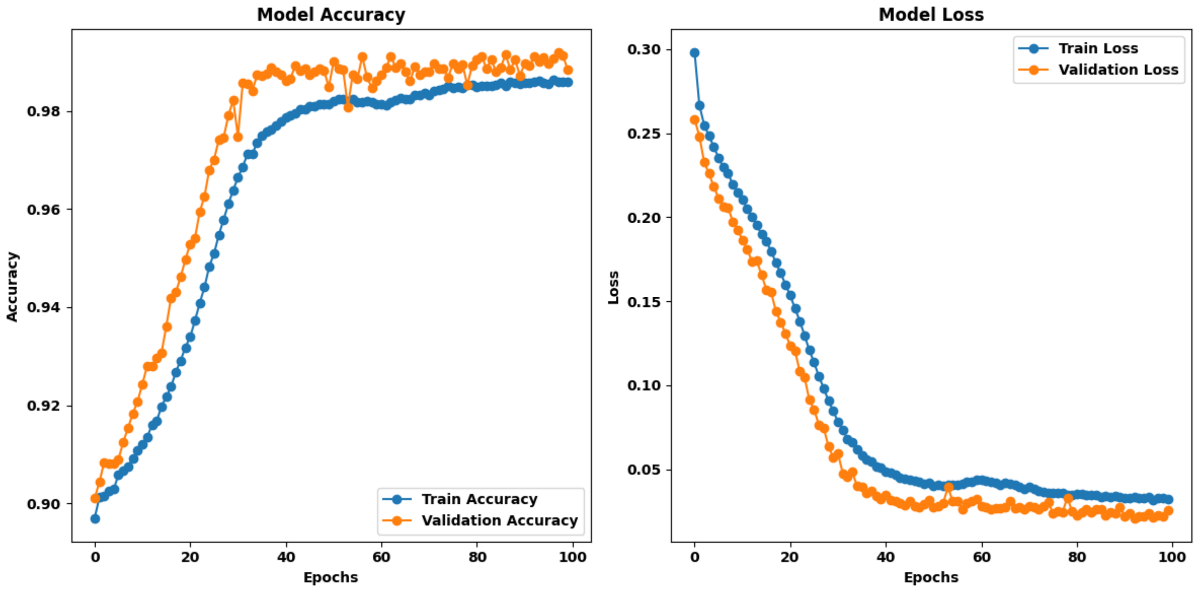

S1 Fig B. Training curves of multi-head neural network (Stage 2: PET Vs Others). Multi head neural network was trained for 100 epochs for PET vs others dataset and its train accuracy was 88.94% and validation accuracy was 90.10%. They began training the model with a training loss of 32.80 and a validation loss of 25.82. Unlike in Stage 1, the training curve exhibited significant oscillation at the initial stages before stabilizing and a smooth curve afterwards while the validation curve exhibited sharp oscillations throughout the process.

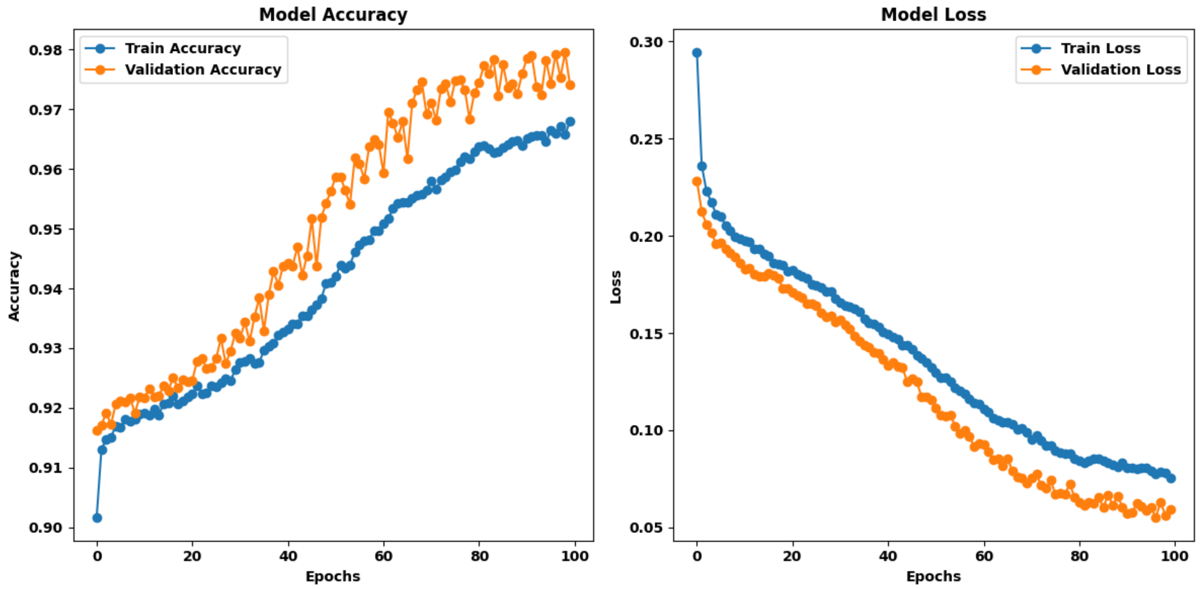

S1 Fig C. Training curves of multi-head neural network (Stage 3: PET Coloured Vs PET Transparent). Comparing PET Coloured and PET Transparent datasets, the multi- head neural network was trained with 100 epochs initial training accuracy of 87.62% and the validation accuracy proved to be 91.62%. A training loss of 36.94 and validation loss of 22.83 was achieved the type of training used in this study. The same, as in Stage 2, the training curve had initial oscillations, but its variation became less in the course of time. The validation curve, on the other hand, continued to oscillate when the model was evaluated because the model's performance was inconsistent.

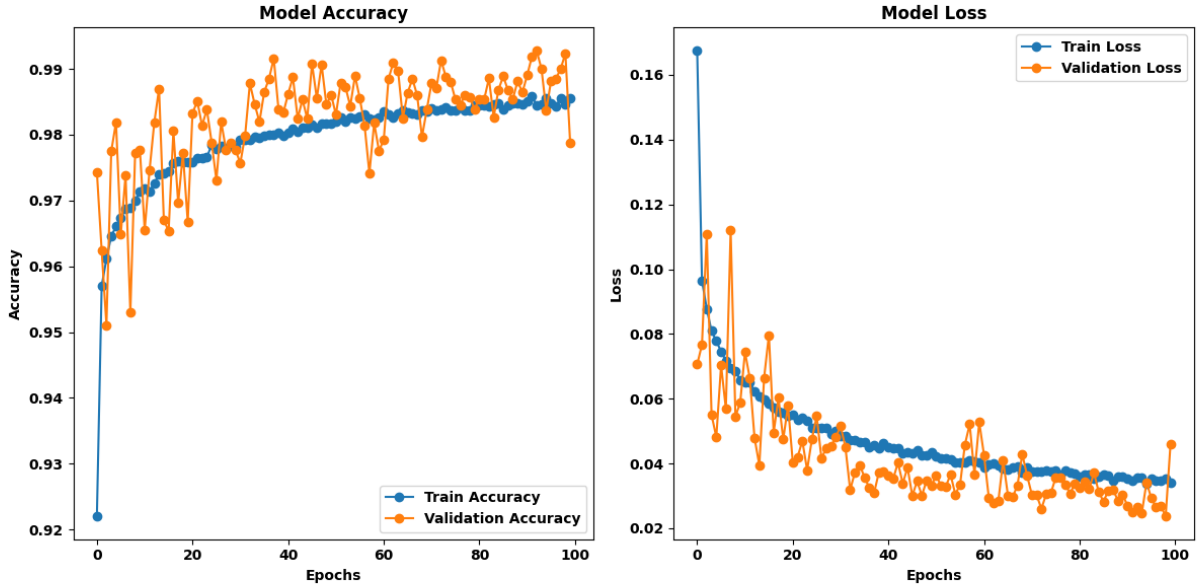

S1 Fig D. Training curves of GRU (Stage 1: PET Clear Vs PET Hazard). The PET Clear vs PET Hazard dataset for 100 epochs resulted in its first training accuracy of 87.08% and a validation accuracy of 97.43%. For the training, the model had a loss of 26.57, and the model had a loss of 7.06 for the validation. During the training stage, the curve increased and decreased multiple times within each epoch, but as epochs progressed, the curve became steadier. Nonetheless, the validation set continued to give infrequent oscillations, and thus indicated the unsuitability of the model for this process.

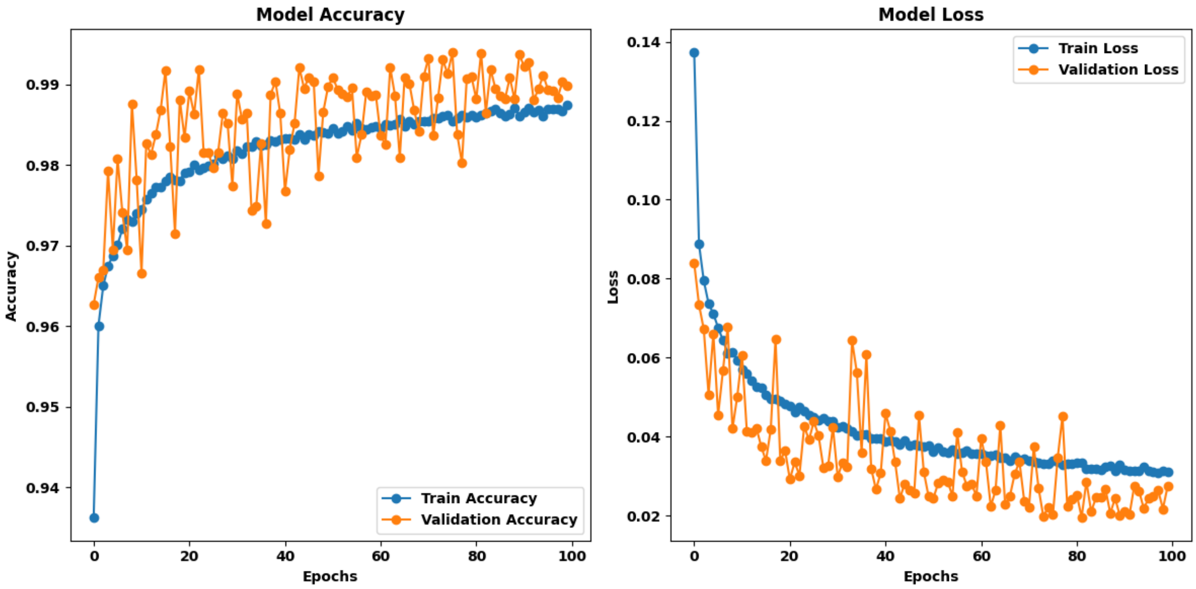

S1 Fig E. Training curves of GRU (Stage 2: PET Vs Others). PET vs others dataset for the GRU model trained over 100 epochs got an initial training accuracy of 89.26 % and the validation accuracy of 96.28 %. Training loss was 21.82, validation loss was 8.39. As in Stage 1, during training phases, the training curve had significant fluctuations during each epoch before it became sS1 Table while on the other hand, validations curve remained quite variable, thus making this model unsuiS1 Table for the given classification task.

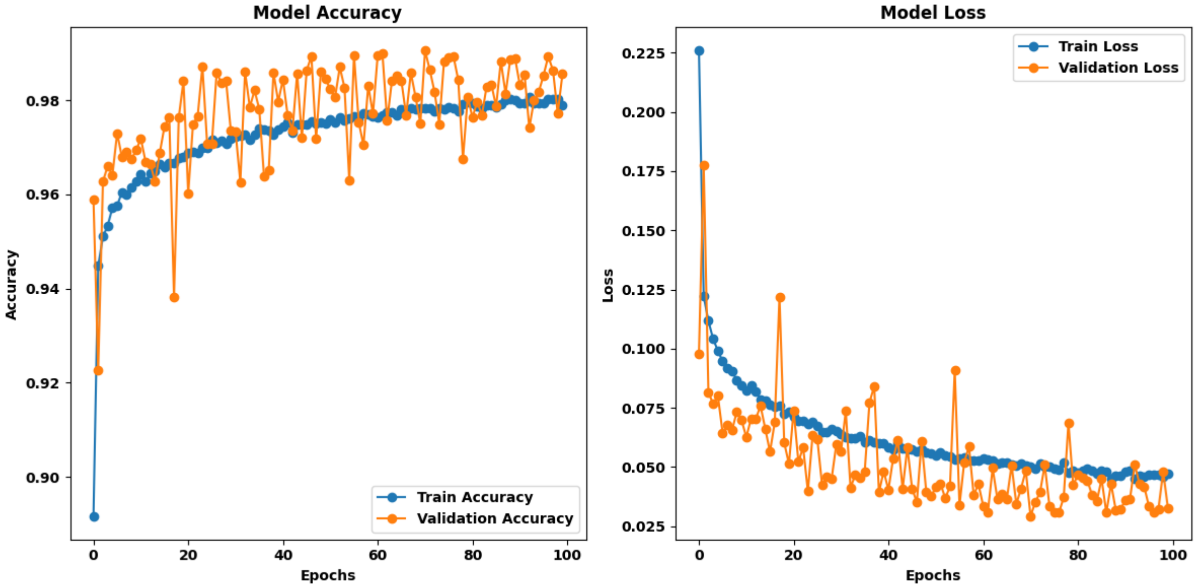

S1 Fig F. Training curves of GRU (Stage 3: PET Coloured Vs PET Transparent). PET Coloured vs PET Transparent dataset, in the GRU model training for 100 epochs the training accuracy that was achieved initially-83.79% and the validation accuracy of 95.88% was obtained. The initial training loss was 32.78 and the validation loss was 9.77. Similarly, in this stage, the training curve showed significant oscillations before the convergence, whereas the validation curve remained irregular, which pointed to the model's inability to provide consistent performance.

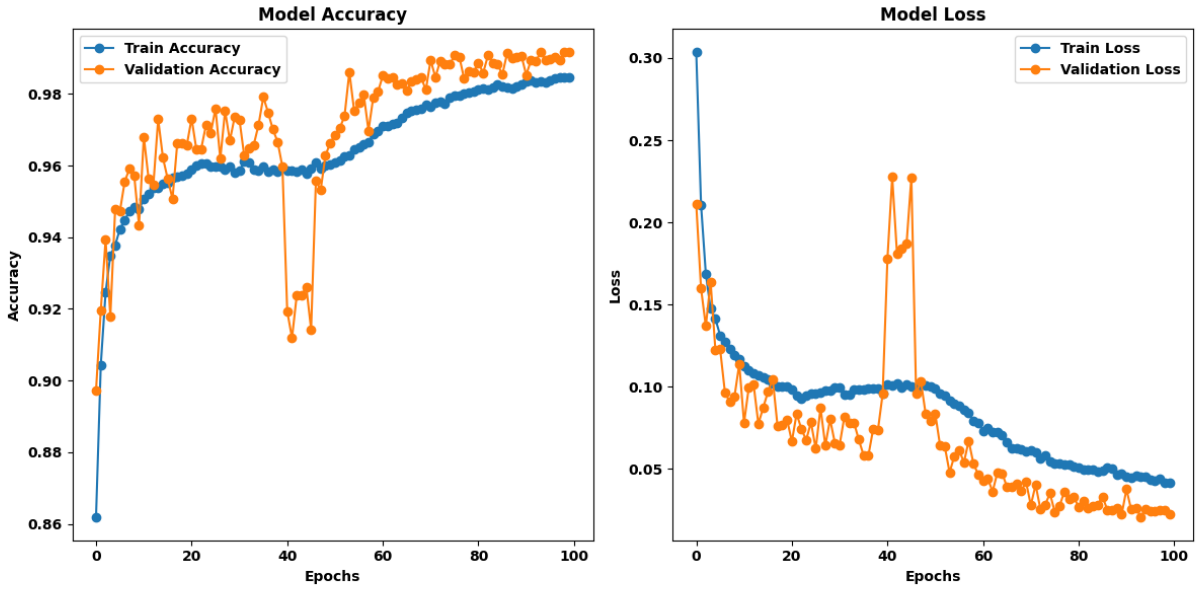

S1 Fig G. Training curves of VGG16 (Stage 1: PET Clear Vs PET Hazard). When the model was trained on PET Clear vs PET Hazard dataset for 100 epochs, it obtained 83.78% training accuracy and 89.72% a validation accuracy. The training loss was obtained as 35.67 While the validations loss was recorded as 21.11. During training, the training data exhibited huge oscillations in the training curve during initial epochs, and later slows down the oscillations. However, the fluctuation recorded in the validation accuracy was not smooth but characterized by recurrent oscillations.

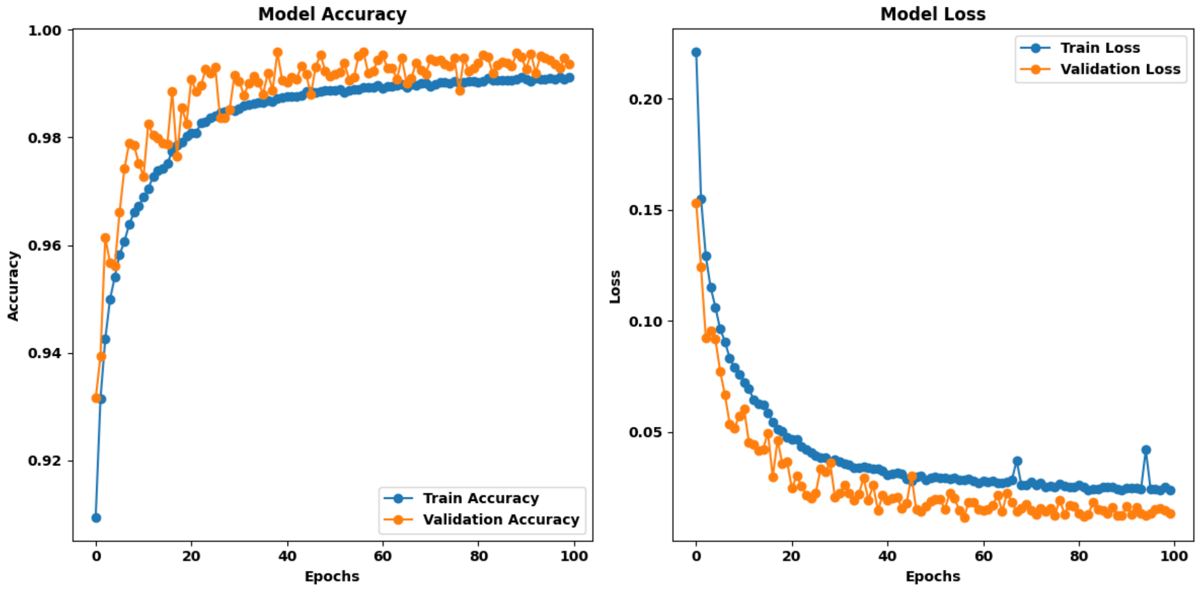

S1 Fig H. Training curves of VGG16 (Stage 2: PET Vs Others). The VGG16 model achieved an initial training accuracy of 89.99% and the validation accuracy of 93.16% in 100 epochs. The training loss was 25.95 while the validation loss was at 15.33. The training curve was visually flattened over vast epoch numbers while the validation curve was irregular, which proved that there could not be steady and consistent performance and generalization found in the model.

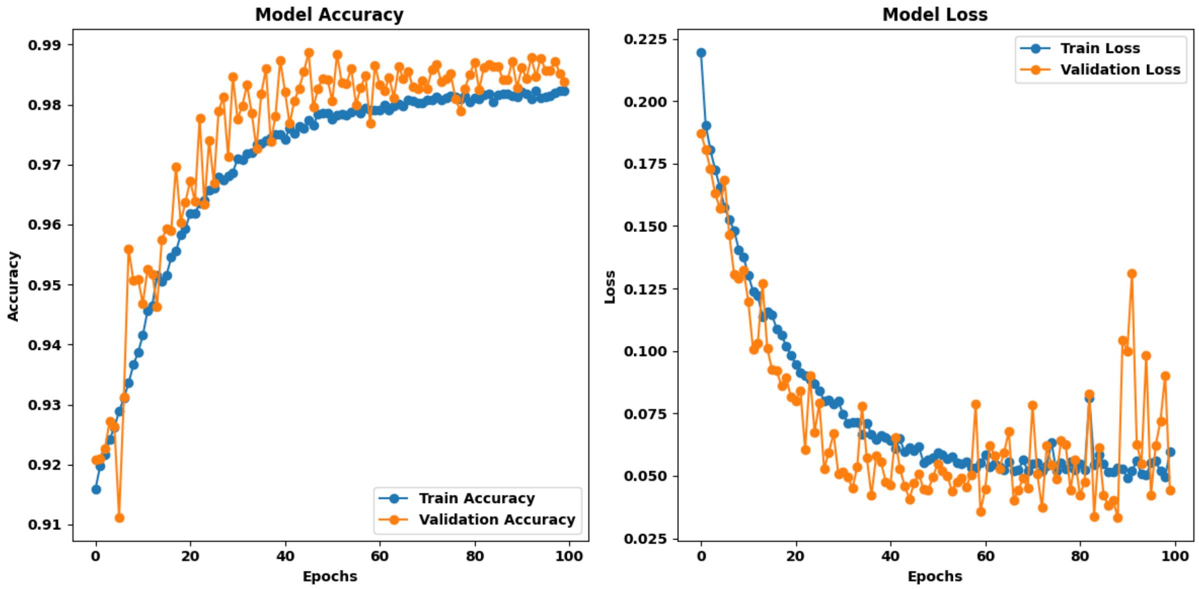

S1 Fig I. Training curves of VGG16 (Stage 3: PET Coloured Vs PET Transparent). On PET Coloured vs PET Transparent dataset the VGG16 model obtained the initial training accuracy of 91.21% and the validation accuracy of 92.08 %. The training loss that was recorded was 24.82 while the validation loss was 18.72. As with the previous stages, while the training curve is more sS1 Table after initial oscillations, validation curve remains irregular which demonstrate fluctuations in the model's validation performance.

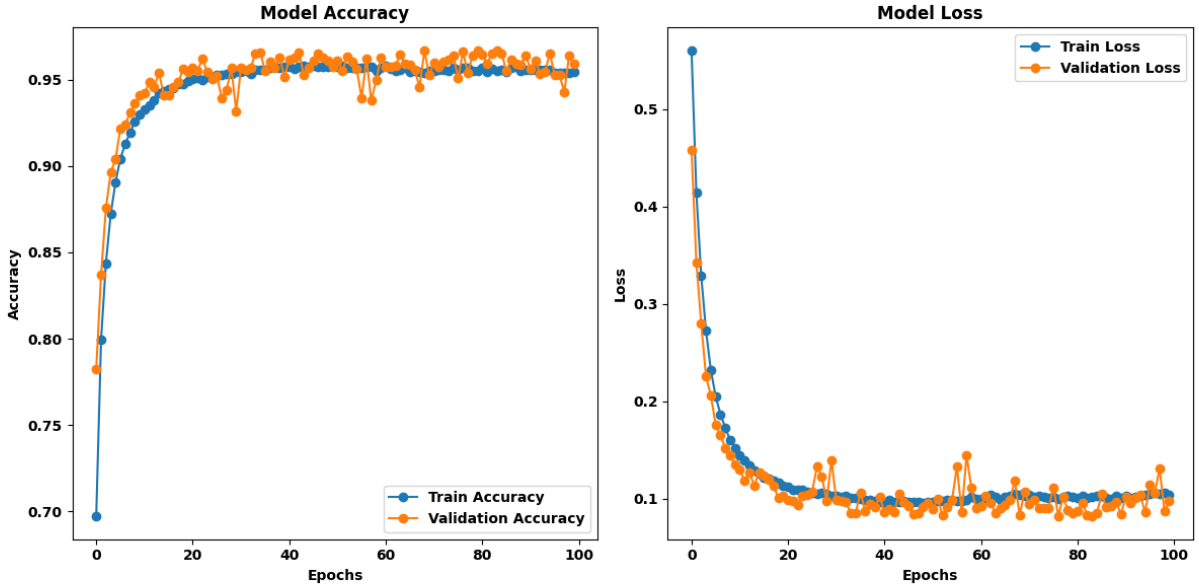

S1 Fig J. Training curves of 1DCNN (Stage 1: PET Clear Vs PET Hazard). Comparing the PET Clear vs PET Hazard dataset for 100 epoch, the initial classifier was able to achieve 63.91 % of training accuracy and 78.24% of validation accuracy. As for the results obtained, the training loss was 61.79 and the validation loss was 45.84. The training curve is nearly flat and gradually turned into a smooth line, different from the wild swings of the validation curve over the training stage.

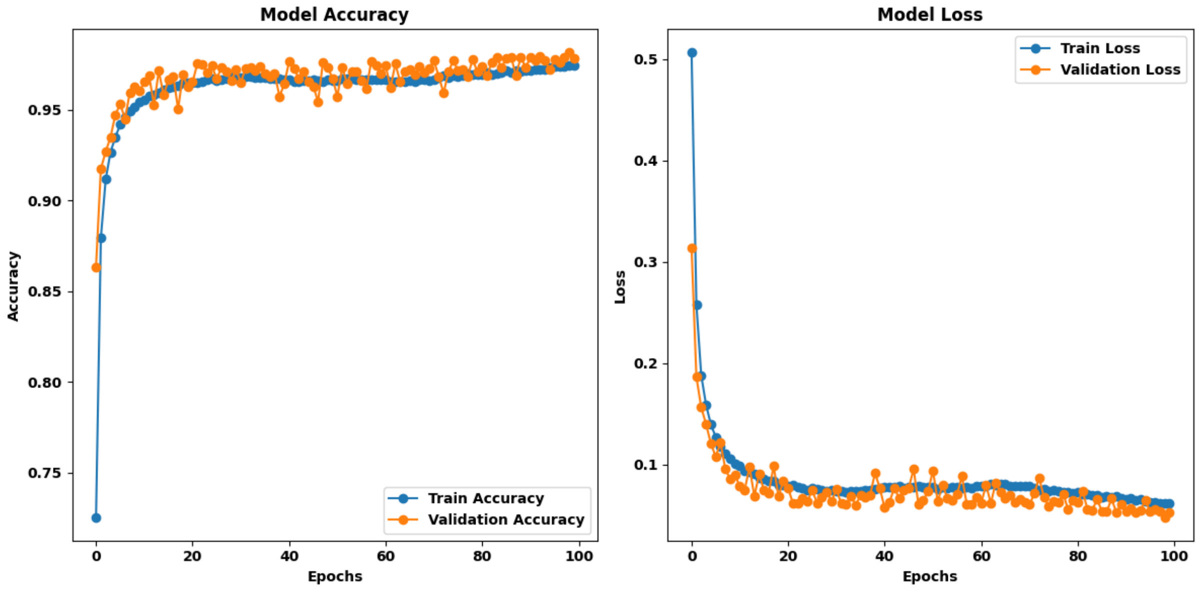

S1 Fig K. Training curves of 1DCNN (Stage 2: PET Vs Others). In comparison between PET and Others dataset the 1D CNN model achieved the initial training accuracy of about 64.82% and the validation accuracy of about 86.35%. The training loss was 59.18 and the validation loss was 31.37. While training error gradually level off up and down, and later became relatively sS1 Table, validation error oscillated up and down for epochs, revealing that the model's validation performance was still unsS1 Table.

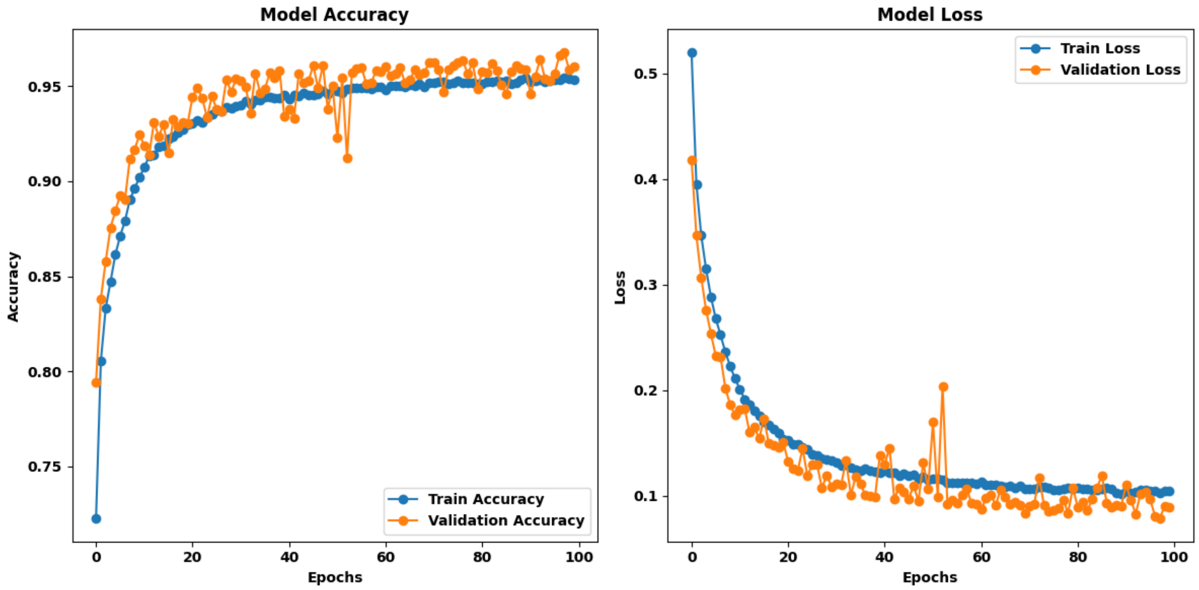

S1 Fig L. Training curves of VGG16 (Stage 3: PET Coloured Vs PET Transparent). The PET Coloured and PET Transparent data and for 1D-CNN, training and validation accuracy of 66.66% and 79.43% respectively were observed. The training loss was 58.31 and validation loss was 41.81. Still, although the training curve reach a steady state after the first oscillations, the validation curve continued to be volatile and remained in this state, suggesting further difficulties regarding model's capabilities to generalize.

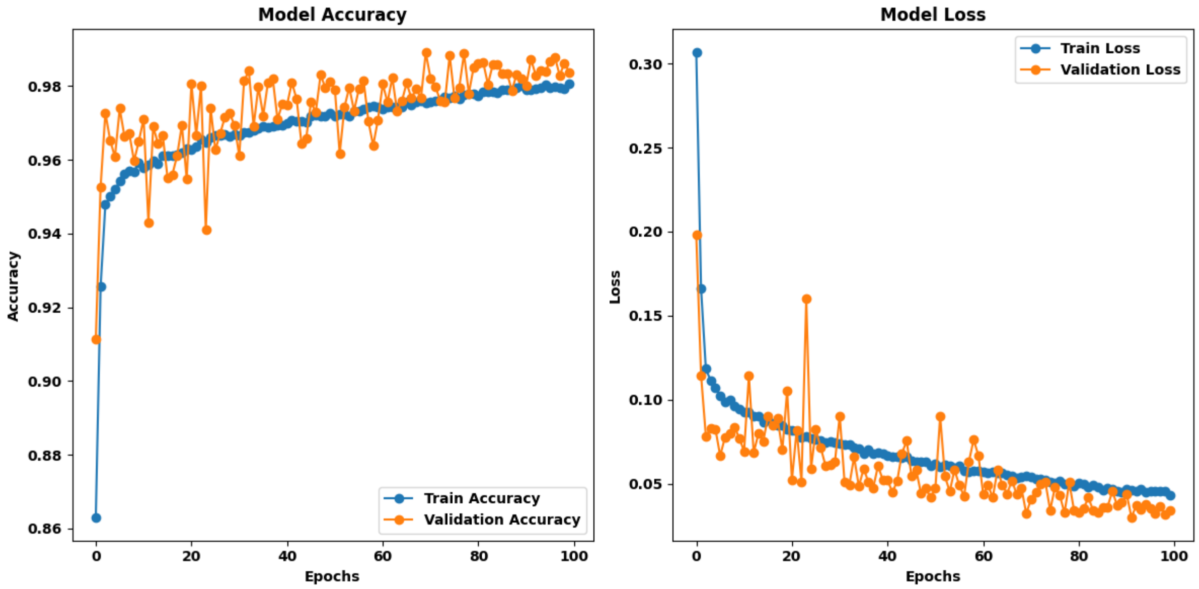

S1 Fig M. Training curves of LSTM (Stage 1: PET Clear Vs PET Hazard). When compared to the PET Hazard and PET Clear dataset for 100 epochs, it obtained an initial training accuracy of 83.68% and the model validation accuracy of 90.13%. In the training phase, the model achieved a training loss of 38.16 and a validation loss of 19.80. Similarly, while the training curve was fluctuating a lot during each epoch and became quite sS1 Table later, the validation curve entities huge variations during epoch, which means that the model has relatively poor ability in generalization for this classification work.

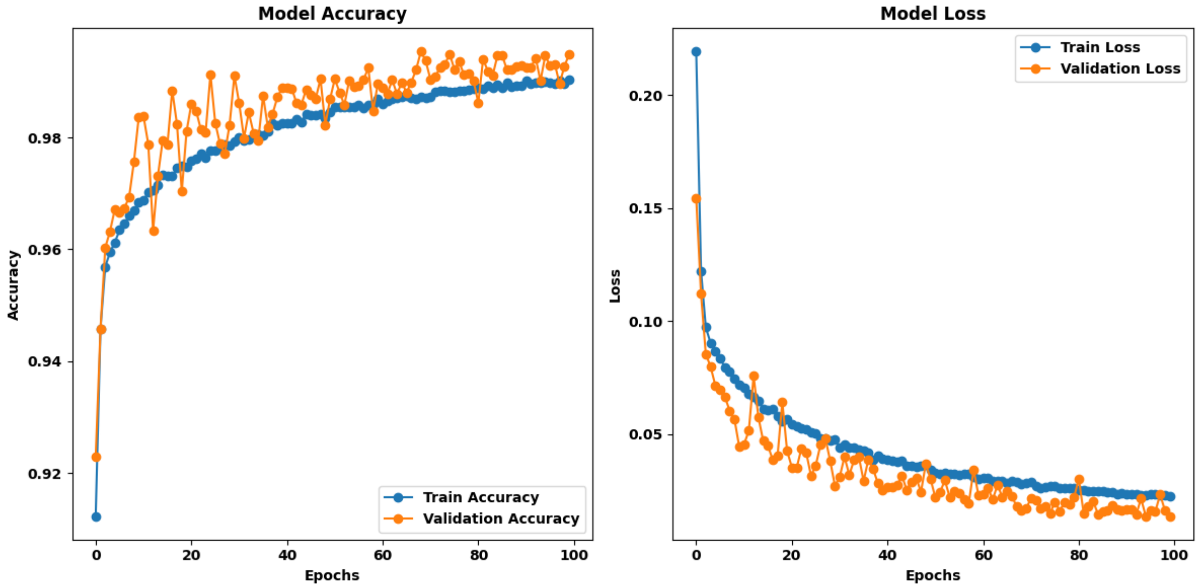

S1 Fig N. Training curves of LSTM (Stage 2: PET Vs Others). The LSTM model achieved the initial training accuracy of 90.16% and the validation accuracy of 92.29% for 100 training epoch. While the training loss variable was 27.89, the validation loss was 15.45. Whilst the training curve was flattening over time, the validation curve was fluctuating tremendously, this meant that there were constant feuds with this model during this stage and hence its unreliability.

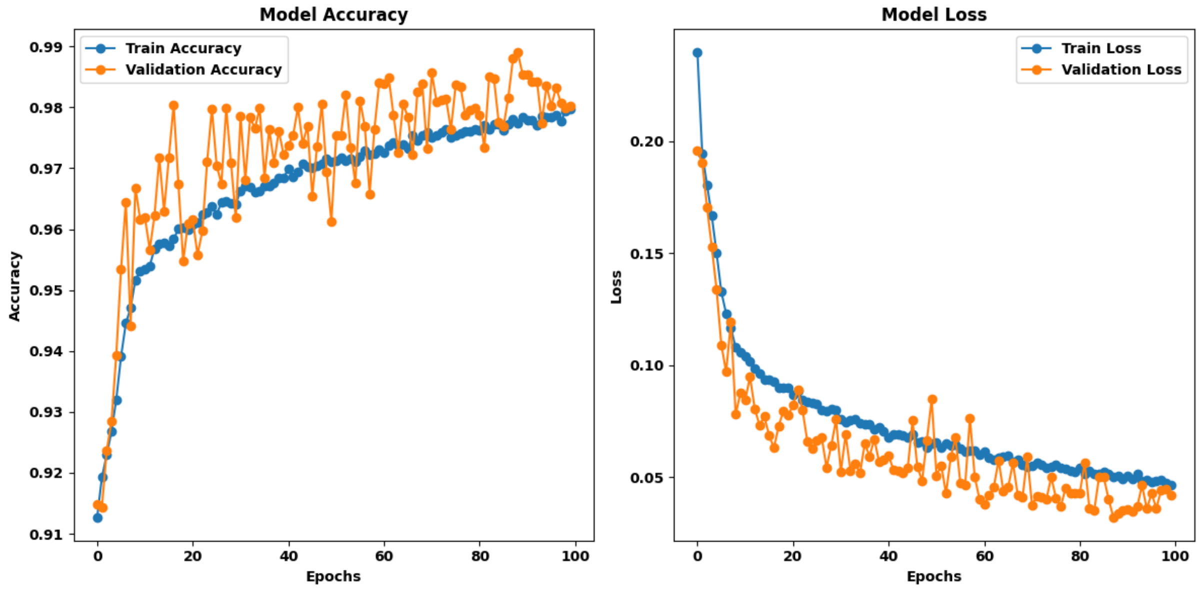

S1 Fig O. Training curves of LSTM (Stage 3: PET Coloured Vs PET Transparent). The LSTM model trained up to 90.55% of accuracy and a cross verified for validation of up to 91.48% of accuracy. The training loss was 29.91 whereas the validation loss was 19.59. As with the previous stages, the training curve leveled out after varying oscillations while the validation curve never became sS1 Table throughout the training phase, which made it a less suiS1 Table model for this task.

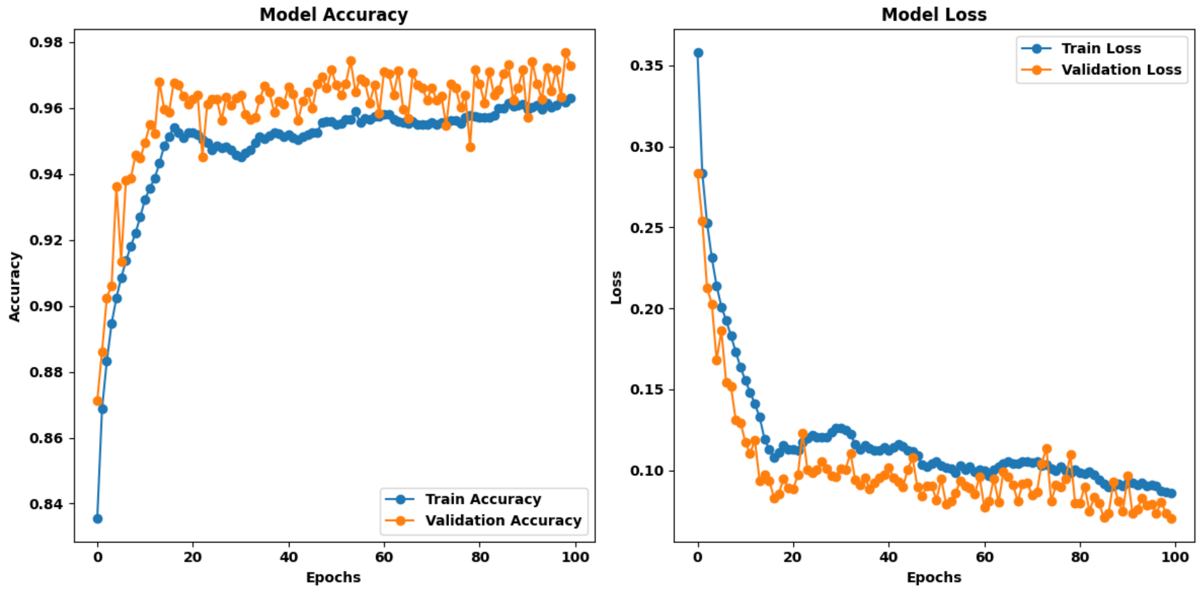

S1 Fig P. Training curves of ResNet (Stage 1: PET Clear Vs PET Hazard). When comparing the PET Clear and PET Hazard dataset for 100 epochs, the model was attained an initial training accuracy of 81.36% and the validation accuracy of 87.14%. The training loss obtained for it was 40.45 while the validation loss was 28.35. Dynamics of training during each epoch, the model's training curve oscillated relatively steeply, but in the end, they became gentler. Nonetheless, the validation curve was volatile, continuing to oscillate.

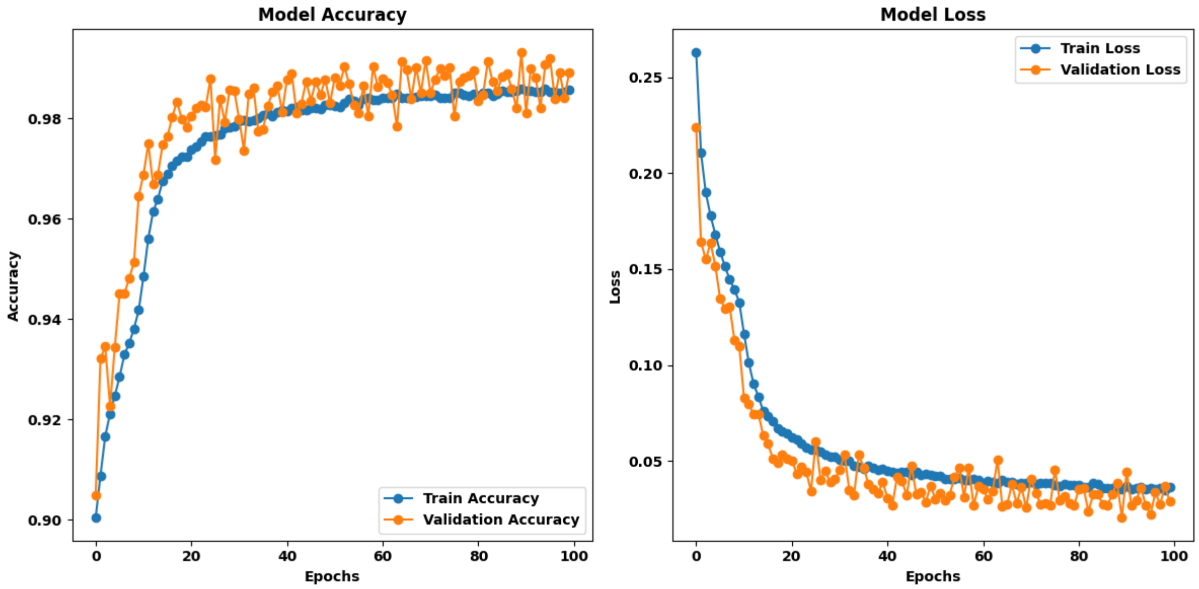

S1 Fig Q. Training curves of ResNet (Stage 2: PET Vs Others). The ResNet after training with 100 of Epoch reached a training accuracy of 89.83% and validation accuracy of 90.49% The training loss was, thereby, obtained as 28.80 while the validation loss was calculated as 22.38. The training process was characterized with significant variations during each epoch while the training curve was fixed at some point. However, as can be seen from the validation curve above, this increases an element of variability and does not necessarily stabilize the model for this classification stage.

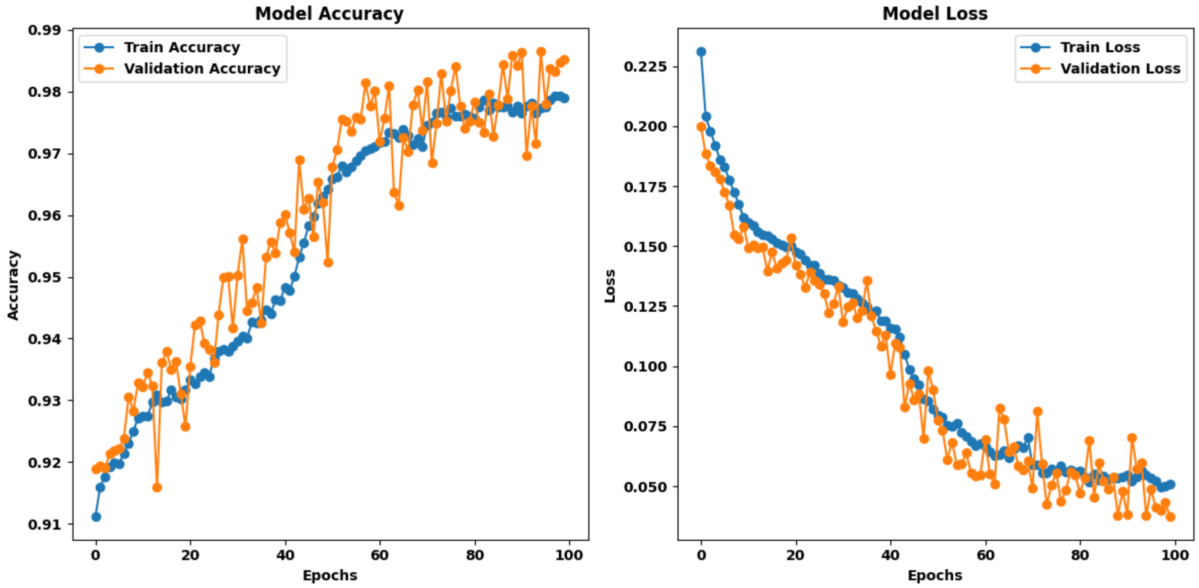

S1 Fig R. Training curves of ResNet (Stage 3: PET Coloured Vs PET Transparent). The ResNet model achieved a training accuracy of 90.43% and a validation accuracy of 91.90% after 100 epochs. The training loss was 25.41 and the validation loss was 19.98. While the training curve shows the oscillation is settled after the first couple of epochs, the validation curve was oscillating even in the later epochs, indicating the model does not generalize well for this stage.

Testing result using confusion matrix

S1 Table A. Performance metric of multi-head neural network (Stage 1: PET Clear Vs PET Hazard).

Metric	PET Clear	PET Hazard
**Accuracy**	98.05	98.05
**Precision**	98.94	95.17
**Recall**	98.78	95.70
**F1 Score**	98.86	95.43
**Specificity**	95.73	98.77

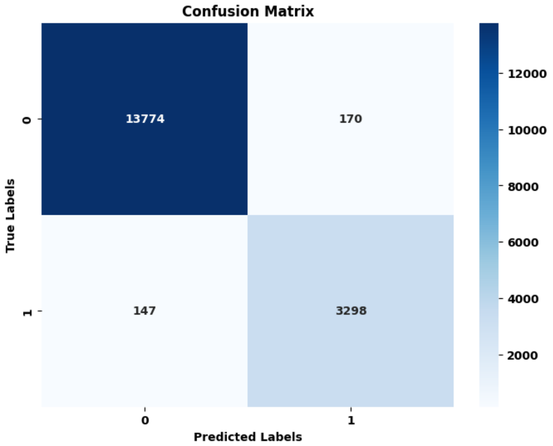

S1 Fig S. Confusion matrix of multi-head neural network (Stage 1: PET Clear Vs PET Hazard). The model accurately predicted 13,774 samples of PET Clear and 3,298 samples of PET Hazard correctly while 170 and 147 were misclassified respectively.

S1 Table B. Performance metric of multi-head neural network (Stage 2: PET Vs Others)

Metric	PET	Others
**Accuracy**	98.85	98.85
**Precision**	99.67	91.97
**Recall**	98.94	97.05
**F1 Score**	99.30	94.36
**Specificity**	97.05	99.04

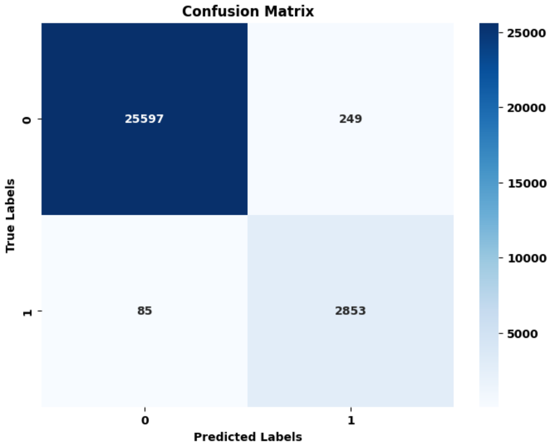

S1 Fig T. Confusion matrix of multi-head neural network (Stage 2: PET Vs Others). There were 25997 instances of PET recognized correctly, 2853 instances of Others also being recognized correctly, with 249 misclassified instances for PET and 85 for Others.

S1 Table C. Performance metric of multi-head neural network (Stage 3: PET Coloured Vs PET Transparent).

Metric	PET Coloured	PET Transparent
**Accuracy**	97.40	97.40
**Precision**	89.05	98.12
**Recall**	83.05	98.73
**F1 Score**	85.84	98.42
**Specificity**	99.82	83.05

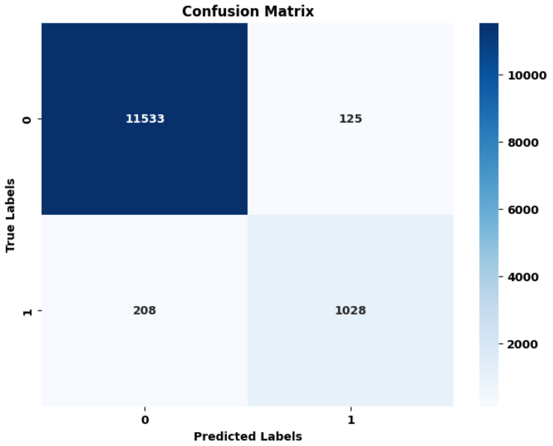

S1 Fig U. Confusion matrix of multi-head neural network (Stage 3: PET Coloured Vs PET Transparent). Between PET Coloured and PET Transparent, PET transparent was classified correctly 11,533 times and PET coloured was classified correctly 1,028 times while PET transparent was misclassified 125 while PET coloured was misclassified 208 times.

S1 Table D. Performance metric of GRU (Stage 1: PET Clear Vs PET Hazard).

Metric	PET Clear	PET Hazard
**Accuracy**	99.69	99.69
**Precision**	96.03	95.63
**Recall**	96.52	96.25
**F1 Score**	95.82	95.94
**Specificity**	96.25	95.63

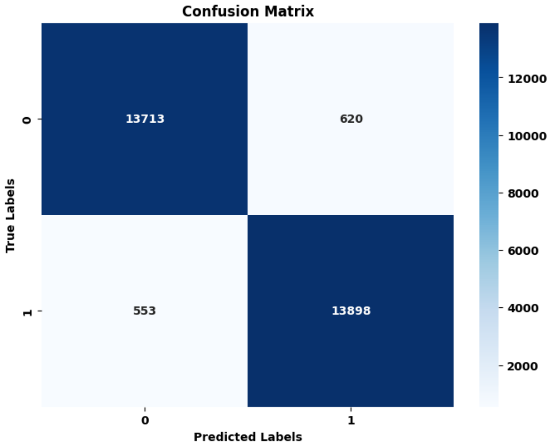

S1 Fig V. Confusion matrix of GRU (Stage 1: PET Clear Vs PET Hazard). The model accurately predicted 13,713 samples of PET Clear and 13,898 samples of PET Hazard correctly while 620 and 553 were misclassified respectively.

S1 Table E. Performance metric of GRU (Stage 2: PET Vs Others).

Metric	PET	Others
**Accuracy**	99.33	99.33
**Precision**	99.21	98.39
**Recall**	98.37	99.38
**F1 Score**	98.79	98.84
**Specificity**	99.42	98.57

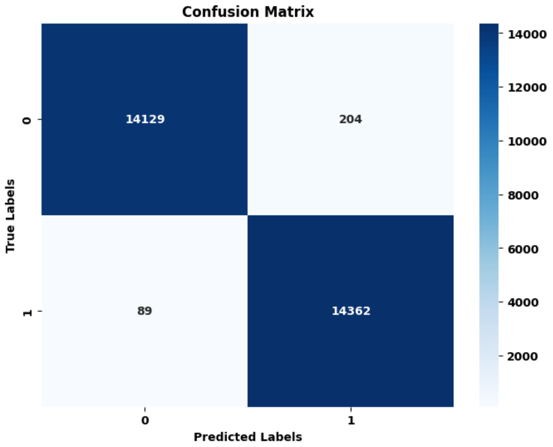

S1 Fig W. Confusion matrix of GRU (Stage 2: PET Vs Others). 14129 instances of PET recognized correctly, 14362 instances of Others also being recognized correctly, with 204 misclassified instances for PET and 89 for Others.

S1 Table F. Performance metric of GRU (Stage 3: PET Coloured Vs PET Transparent)

Metric	PET Coloured	PET Transparent
**Accuracy**	98.64	98.64
**Precision**	99.13	98.07
**Recall**	98.05	99.19
**F1 Score**	98.58	98.63
**Specificity**	98.05	97.95

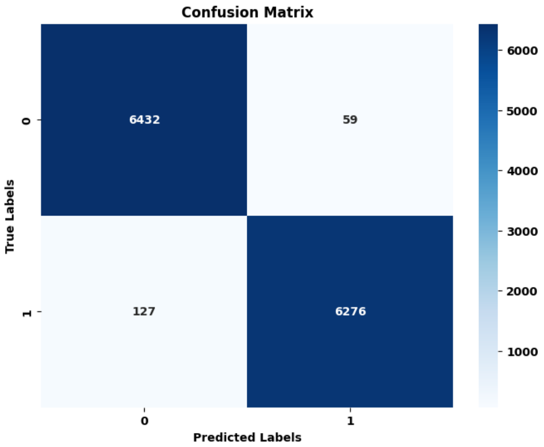

S1 Fig X. Confusion matrix of GRU (Stage 3: PET Coloured Vs PET Transparent). PET transparent was classified correctly 6,432 times and PET coloured was classified correctly 6,276 times while PET transparent was misclassified 59 while PET coloured was misclassified 127 times.

S1 Table G. Performance metric of VGG16 (Stage 1: PET Clear Vs PET Hazard).

Metric	PET Clear	PET Hazard
**Accuracy**	99.16	99.16
**Precision**	99.32	98.36
**Recall**	99.55	97.17
**F1 Score**	99.44	97.76
**Specificity**	99.17	99.30

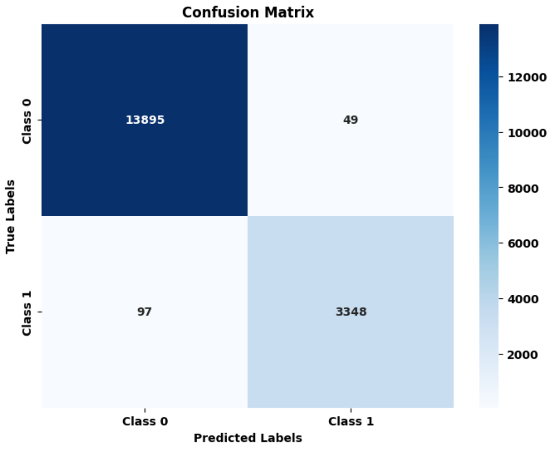

S1 Fig Y. Confusion matrix of VGG16 (Stage 1: PET Clear Vs PET Hazard). The model accurately predicted 13,895 samples of PET Clear and 3,348 samples of PET Hazard correctly while 49 and 97 were misclassified respectively.

S1 Table H. Performance metric of VGG16 (Stage 2: PET Vs Others).

Metric	PET	Others
**Accuracy**	99.55	99.55
**Precision**	99.72	96.25
**Recall**	99.56	97.43
**F1 Score**	99.64	96.83
**Specificity**	97.43	99.72

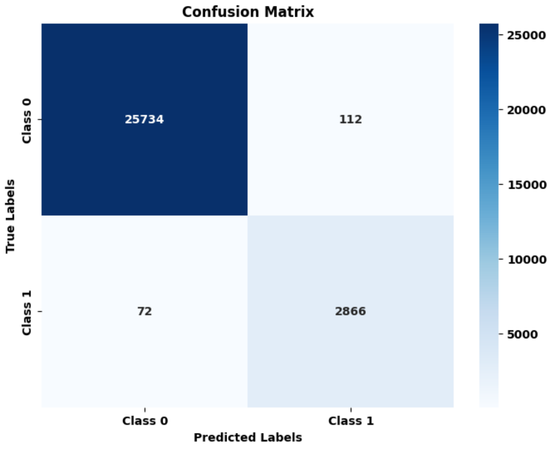

S1 Fig Z. Confusion matrix of VGG16 (Stage 2: PET Vs Others). 25734 instances of PET recognized correctly, 2866 instances of Others also being recognized correctly, with 112 misclassified instances for PET and 72 for Others.

S1 Table I. Performance metric of VGG16 (Stage 3: PET Coloured Vs PET Transparent).

Metric	PET Coloured	PET Transparent
**Accuracy**	97.65	97.65
**Precision**	95.77	98.56
**Recall**	86.83	99.59
**F1 Score**	90.89	99.07
**Specificity**	98.62	86.83

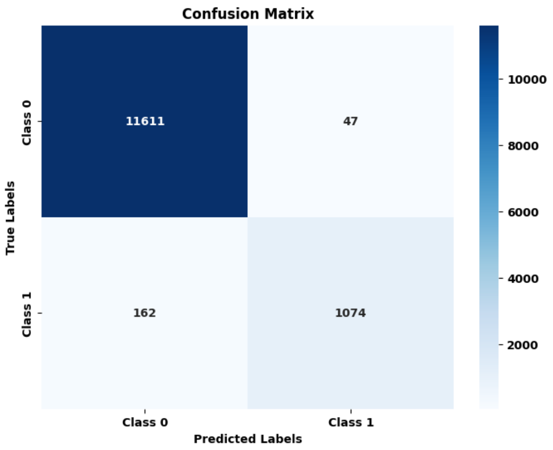

S1 Fig AA. Confusion matrix of VGG16 (Stage 3: PET Coloured Vs PET Transparent). PET transparent was classified correctly 11,611 times and PET coloured was classified correctly 1,074 times while PET transparent was misclassified 47 while PET coloured was misclassified 162 times.

S1 Table J. Performance metric of 1DCNN (Stage 1: PET Clear Vs PET Hazard)

Metric	PET Clear	PET Hazard
**Accuracy**	99.23	99.23
**Precision**	98.70	93.39
**Recall**	93.07	98.76
**F1 Score**	95.84	95.96
**Specificity**	98.76	93.13

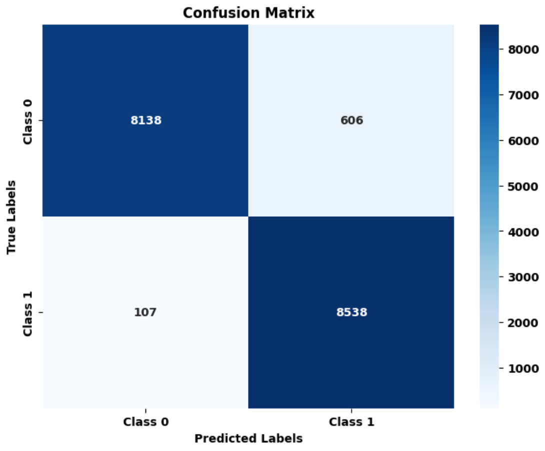

S1 Fig BB. Confusion matrix of 1DCNN (Stage 1: PET Clear Vs PET Hazard). The model accurately predicted 8138 samples of PET Clear and 8538 samples of PET Hazard correctly while 606 and 107 were misclassified respectively.

S1 Table K. Performance metric of 1DCNN (Stage 2: PET Vs Others)

Metric	PET	Others
**Accuracy**	98.61	98.61
**Precision**	96.99	98.65
**Recall**	98.66	96.96
**F1 Score**	97.82	97.80
**Specificity**	96.96	98.66

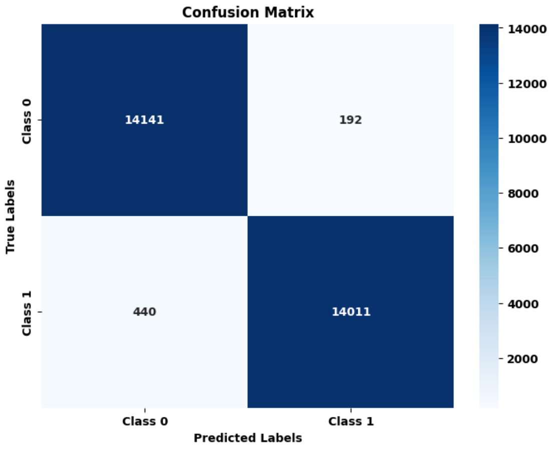

S1 Fig CC. Confusion matrix of 1DCNN (Stage 2: PET Vs Others). 14141 instances of PET recognized correctly, 14011 instances of Others also being recognized correctly, with 192 misclassified instances for PET and 440 for Others.

S1 Table L. Performance metric of 1DCNN (Stage 3: PET Coloured Vs PET Transparent)

Metric	PET Coloured	PET Transparent
**Accuracy**	96.23	96.23
**Precision**	95.05	97.06
**Recall**	97.08	95.01
**F1 Score**	96.06	96.03
**Specificity**	95.01	97.08

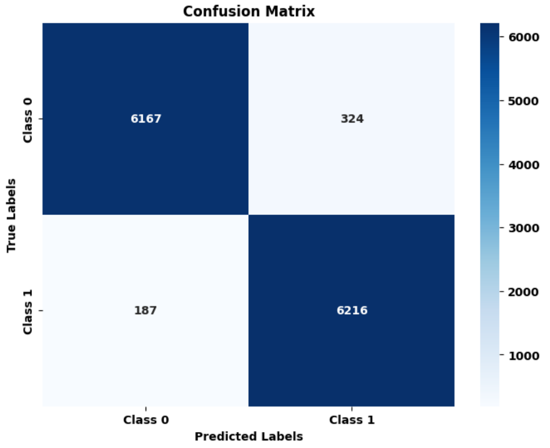

S1 Fig DD. Confusion matrix of 1DCNN (Stage 3: PET Coloured Vs PET Transparent). PET transparent was classified correctly 6,167 times and PET coloured was classified correctly 6,216 times while PET transparent was misclassified 324 while PET coloured was misclassified 187 times.

S1 Table M. Performance metric of LSTM (Stage 1: PET Clear Vs PET Hazard).

Metric	PET Clear	PET Hazard
**Accuracy**	98.35	98.35
**Precision**	98.79	96.23
**Recall**	99.17	95.53
**F1 Score**	98.98	95.88
**Specificity**	95.45	98.89

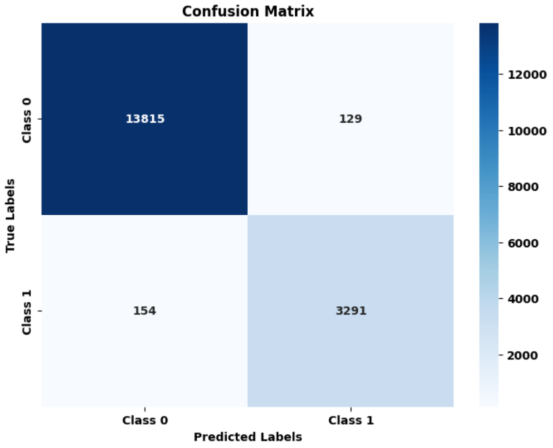

S1 Fig EE. Confusion matrix of LSTM (Stage 1: PET Clear Vs PET Hazard). The model accurately predicted 13,815 samples of PET Clear and 3,291 samples of PET Hazard correctly while 129 and 154 were misclassified respectively.

S1 Table N. Performance metric of LSTM (Stage 2: PET Vs Others).

Metric	PET	Others
**Accuracy**	99.49	99.49
**Precision**	99.60	97.45
**Recall**	99.67	97.45
**F1 Score**	99.63	97.45
**Specificity**	97.45	99.72

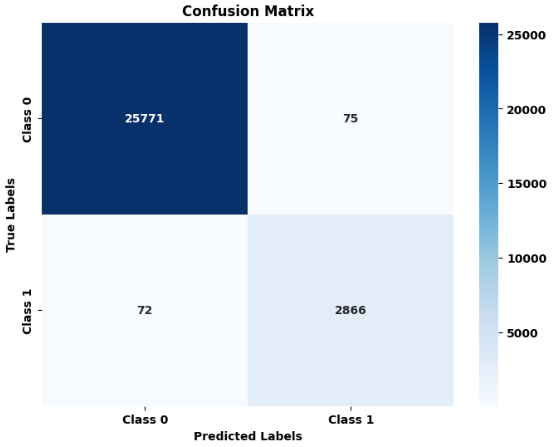

S1 Fig FF. Confusion matrix of LSTM (Stage 2: PET Vs Others). 25771 instances of PET recognized correctly, 2866 instances of Others also being recognized correctly, with 75 misclassified instances for PET and 72 for Others.

S1 Table O. Performance metric of LSTM (Stage 3: PET Coloured Vs PET Transparent).

Metric	PET Coloured	PET Transparent
**Accuracy**	97.37	97.37
**Precision**	90.15	98.72
**Recall**	89.15	98.74
**F1 Score**	89.65	98.73
**Specificity**	98.85	89.15

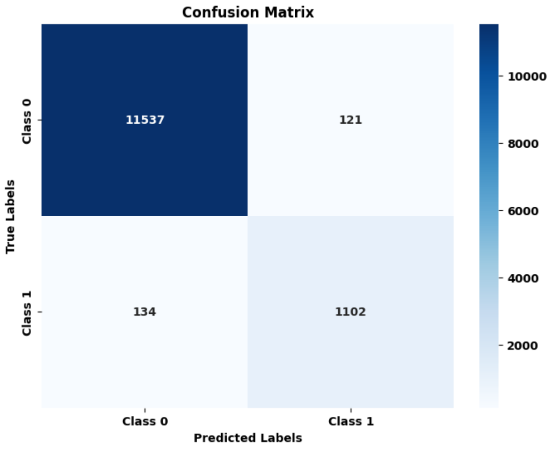

S1 Fig GG. Confusion matrix of LSTM (Stage 3: PET Coloured Vs PET Transparent). PET transparent was classified correctly 11,537 times and PET coloured was classified correctly 1,102 times while PET transparent was misclassified 121 while PET coloured was misclassified 134 times.

S1 Table P. Performance metric of ResNet (Stage 1: PET Clear Vs PET Hazard).

Metric	PET Clear	PET Hazard
**Accuracy**	97.14	97.14
**Precision**	97.45	95.47
**Recall**	98.95	90.73
**F1 Score**	98.19	93.05
**Specificity**	90.56	97.96

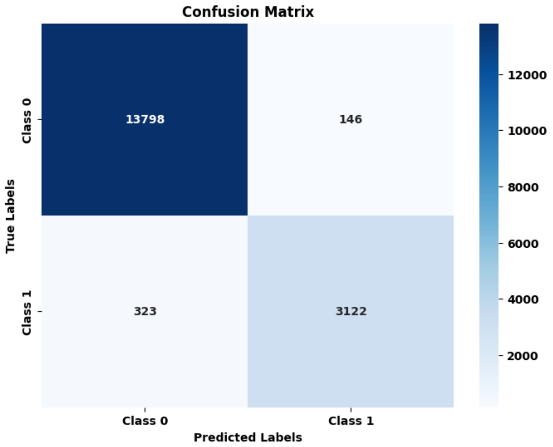

S1 Fig HH. Confusion matrix of ResNet (Stage 1: PET Clear Vs PET Hazard). The model accurately predicted 13,798 samples of PET Clear and 3,122 samples of PET Hazard correctly while 146 and 323 were misclassified respectively.

S1 Table Q. Performance metric of ResNet (Stage 2: PET Vs Others)

Metric	PET	Others
Accuracy	98.32	98.32
Precision	99.35	94.03
Recall	99.29	95.37
F1 Score	99.32	94.69
Specificity	95.47	99.45

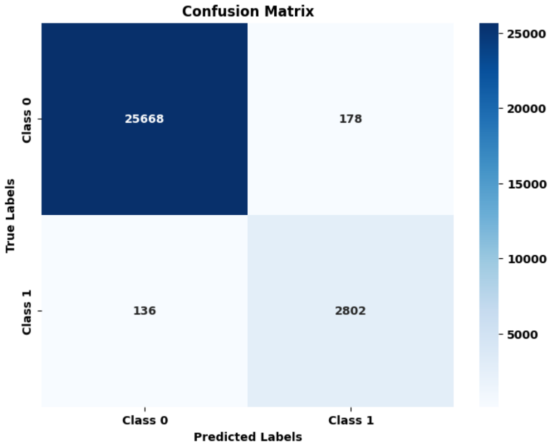

S1 Fig II. Confusion matrix of ResNet (Stage 2: PET Vs Others). 25668 instances of PET recognized correctly, 2802 instances of Others also being recognized correctly, with 178 misclassified instances for PET and 136 for Others.

S1 Table R. Performance metric of ResNet (Stage 3: PET Coloured Vs PET Transparent).

Metric	PET Coloured	PET Transparent
Accuracy	98.57	98.57
Precision	96.45	98.67
Recall	87.64	99.66
F1 Score	91.79	99.16
Specificity	98.73	87.74

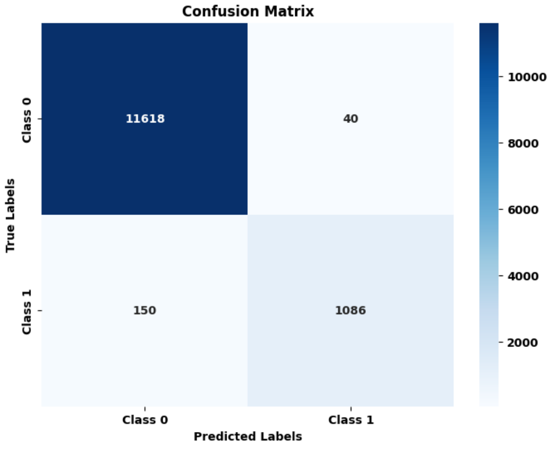

S1 Fig JJ. Confusion matrix of ResNet (Stage 3: PET Coloured Vs PET Transparent). PET transparent was classified correctly 11,618 times and PET coloured was classified correctly 1,086 times while PET transparent was misclassified 40 while PET coloured was misclassified 150 times.
